# The WOX family transcriptional regulator SlLAM1 controls compound leaf and floral organ development in *Solanum lycopersicum*

**DOI:** 10.1093/jxb/eraa574

**Published:** 2020-12-05

**Authors:** Chaoqun Wang, Baolin Zhao, Liangliang He, Shaoli Zhou, Ye Liu, Weiyue Zhao, Shiqi Guo, Ruoruo Wang, Quanzi Bai, Youhan Li, Dongfa Wang, Qing Wu, Yuanfan Yang, Yu Liu, Million Tadege, Jianghua Chen

**Affiliations:** 1 CAS Key Laboratory of Tropical Plant Resources and Sustainable Use, CAS Center for Excellence for Molecular Plant Sciences, Xishuangbanna Tropical Botanical Garden, Chinese Academy of Sciences, Kunming, Yunnan, China; 2 University of Chinese Academy of Sciences, Beijing, China; 3 School of Life Sciences, University of Science and Technology of China, Hefei, China; 4 School of Ecology and Environmental Sciences, Yunnan University, Kunming, China; 5 Department of Plant and Soil Sciences, Institute for Agricultural Biosciences, Oklahoma State University, 3210 Sam Noble Parkway, Ardmore, OK, USA; 6 University College Dublin, Ireland

**Keywords:** LAM1, leaf blade outgrowth, leaf development, secondary leaflet initiation, *Solanum lycopersicum*, tomato, WOX1

## Abstract

Plant-specific WOX family transcription factors play important roles ranging from embryogenesis to lateral organ development. The WOX1 transcription factors, which belong to the modern clade of the WOX family, are known to regulate outgrowth of the leaf blade specifically in the mediolateral axis; however, the role of WOX1 in compound leaf development remains unknown. Phylogenetic analysis of the whole WOX family in tomato (*Solanum lycopersicum*) indicates that there are 10 members that represent the modern, intermediate, and ancient clades. Using phylogenetic analysis and a reverse genetic approach, in this study we identified *SlLAM1* in the modern clade and examined its function and tissue-specific expression pattern. We found that knocking out *SlLAM1* via CRISPR/Cas9-mediated genome editing led to narrow leaves and a reduced number of secondary leaflets. Overexpression of tomato *SlLAM1* could rescue the defects of the tobacco *lam1* mutant. Anatomical and transcriptomic analyses demonstrated that floral organ development, fruit size, secondary leaflet initiation, and leaf complexity were altered due to loss-of-function of *SlLAM1*. These findings demonstrate that tomato *SlLAM1* plays an important role in the regulation of secondary leaflet initiation, in addition to its conserved function in blade expansion.

## Teaser

The LAM1 transcription factor regulates expansion of primary leaflets in the compound leaves of tomato, and also affects floral organ development, fruit size, and initiation of secondary leaflets.

## Introduction

Leaves and floral organs are typical lateral organs and originate from the flanks of the shoot apical meristem (SAM) and the floral meristem, respectively. These determinate lateral organs possess inherent polarity, with the adaxial side of the lateral organ primordium being the side closest to the meristem and the abaxial side being away from it (Eshed *et al.*, 2001; Bowman *et al.*, 2002). The HD-ZIPIII, KANADI, YABBY, MYB, and LOB domain proteins together with small RNAs, KNOX, and WUSCHEL-related homeobox (WOX) proteins play important roles in this process in plants (Laux *et al.*, 1996; Waites *et al.*, 1998; Kerstetter *et al.*, 2001; Husbands *et al.*, 2009; Ikeda *et al.*, 2009; Moon and Hake, 2011; Sakakibara *et al.*, 2014; Youngstrom *et al.*, 2019). The WOX family, a large group of transcription factors, belongs to the homeodomain (HD) super family that has a typical DNA-binding domain of ~60 amino acids (Gehring *et al.*, 1994; van der Graaff *et al.*, 2009; Burglin and Affolter, 2016). The WOX homeodomain (WOX HD) is slightly larger (~65–70 aa) due to extension at the C-terminus of the HD (Haecker *et al.*, 2004; van der Graaff *et al.*, 2009; Tadege *et al.*, 2011; Catarino *et al.*, 2016; Romani *et al.*, 2018). Previous reports have shown that *WOX* genes can be divided into three clades based on their phylogenetic relationships and conserved domains, namely the ancient clade, the intermediate clade, and the WUS/modern clade (Haecker *et al.*, 2004; van der Graaff *et al.*, 2009). The WOX transcription factors in Arabidopsis have four main specific motifs in addition to the HD that is conserved in all WOX family members, and these are the WUS box, the EAR-like motif, the STF box, and the MAEWEST/WOX4 box (Haecker *et al.*, 2004; van der Graaff *et al.*, 2009; Ikeda *et al.*, 2009; Tadege *et al.*, 2011). Members of the WUS/modern clade have a particular WUS box near the C-terminus that is required for repressive activity, and this distinguishes the modern clade members from the rest of the WOX family (Haecker *et al.*, 2004; Ikeda *et al.*, 2009; Lin *et al.*, 2013). The other three motifs are found in some specific subclades of the WUS clade. An EAR-like motif is found at the C-terminus of the WUS, WOX5, and WOX7 homologues comprising the WUS and WOX5 subclades (Vandenbussche *et al.*, 2009), and this might contribute to repressive activity, but its contribution is dispensable for at least the WUS function (Ikeda *et al.*, 2009). The MAEWEST/WOX4 box is located at the N-terminus of the HD and exists only in the WOX1 and WOX4 homologues, but its function is unknown (Costanzo *et al.*, 2014). The STF box is found at the C-terminus of only WOX1 and WOX6 homologues and has been shown to be required for the STF (STENOFOLIA) repressive function in leaf and flower development (Zhang *et al.*, 2014).

In Arabidopsis, the *wox1* single-mutant has no discernible phenotype (Haecker *et al.*, 2004; Vandenbussche *et al.*, 2009) and *WOX3* (also called *PRS*) is required for development of lateral stipules in leaves and lateral sepals and stamens in flowers (Matsumoto and Okada, 2001; Shimizu *et al.*, 2009), but *wox1 prs* double-mutants display narrow leaves and floral organs that are affected in lateral expansion of the leaf blade and in fusion of petals (Vandenbussche *et al.*, 2009; Nakata *et al.*, 2012). This indicates that WOX1 and PRS redundantly regulate the expansion of lateral organs including leaves and flowers. However, this redundancy appears to be specific to Arabidopsis because WOX1 homologues in other eudicot species display strong leaf and flower phenotypes as single-mutants. The WOX1 homologue genetic mutants *lam1* in *Nicotiana sylvestris*, *maw* (*maewest*) in petunia, *stf* in *Medicago*, and *lath* (*lathyroides*) in pea show narrow leaf blades and petals (McHale, 1992; Vandenbussche *et al.*, 2009; Tadege *et al.*, 2011; Zhuang *et al.*, 2012), indicating non-redundant WOX1 function in the expansion of lateral organs in these species. In addition, the *Medicago WOX3* homologue mutant *loose flower* (*lfl*) is affected in flower development but not in outgrowth of the leaf blade, and the *stf lfl* double-mutant is identical to *stf* in the leaf-blade phenotype (Niu*et al*., 2015), indicating that LFL/MtWOX3 has no redundant function with STF in blade development. Interestingly, in all of the *WOX1* homologue mutants, the growth defects appear to be specific to the medial-lateral axis and are variable in strength without a significant effect on leaf length and complexity. For example, the *lam1* mutant is extremely narrow in width with almost no blade tissue, but the blade length appears normal (McHale, 1992). The *lam1* mutant is also non-bolting and non-flowering under standard growth conditions (McHale, 1992; Tadege *et al.*, 2011). Similarly, the *wox1 prs*, *maw*, *stf*, and *lath* mutants do not display obvious proximal-distal defects, but the blade phenotypes are relatively weak compared to *lam1*, and they flower normally. Arabidopsis, *N. sylvestris*, and petunia have simple leaves, which makes it difficult to evaluate the effect of *WOX1* and its homologues on leaf complexity; in contrast, *Medicago* and pea have compound leaves, but the trifoliate identity in *stf* mutants (Tadege *et al.*, 2011) and multifoliate identity in *lath* mutants (Zhuang *et al.*, 2012) are indistinguishable from their respective wild-types under standard growth conditions. In addition, both *stf* and *lam1* (induced to flower by high temperature) mutants are female-sterile due to defective ovule development, but the *wox1 prs*, *maw*, and *lath* mutants are fertile, albeit with reduced fertility (Vandenbussche *et al.*, 2009; Nakata *et al.*, 2012; Zhuang *et al.*, 2012). These observations indicate that the function of *WOX1* and its homologues is generally conserved in mediating lateral outgrowth, but also show that there is considerable specificity in the different eudicot species examined, suggesting a dynamic role.

*WOX1* homologues are found only in eudicots and the ancestral species *Amborella trichopoda*, and not in monocots and other taxa (Vandenbussche *et al.*, 2009; Tadege *et al.*, 2011; Zhang *et al.*, 2014). In monocots, leaf-blade expansion is controlled by *WOX3* homologues. In maize, the *WOX3* homologues *NARROW SHEATH 1* and *2* (*NS1* and *NS2*) redundantly regulate blade outgrowth, and the double-mutant shows a strong blade ablation phenotype (Nardmann *et al.*, 2004). Similarly in rice, the *NARROW LEAF2* (*NAL2*) and *NAL3* genes encode the WUSCHEL-related homeobox3 (OsWOX3A) protein (Cho *et al.*, 2013; Ishiwata *et al.*, 2013), and the *nal2 nal3* double-mutant shows strong pleotropic phenotypes in leaf, spikelet, tiller, and lateral root development (Cho *et al.*, 2013). Likewise, *NARROW LEAFED DWARF1* (*NLD1*), encoding a WUSCHEL-related homeobox3 protein, controls the development of lateral organs in barley, as demonstrated by the narrow leaf-blade phenotype of the *nld1* mutant (Yoshikawa *et al.*, 2016). But these monocots have simple leaves, and it is unclear if *WOX3* homologues control leaf complexity. In addition, *WOX1/6* orthologs are expressed in different stages of young leaf primordia but are absent from the SAM, and are also enriched at the adaxial–abaxial boundary layer (Vandenbussche *et al.*, 2009; Tadege *et al.*, 2011; Nakata *et al.*, 2012; Zhuang *et al.*, 2012;Niu *et al.*, 2018). In monocots, expression of *WOX3* orthologs is enriched in shoot meristems and in the marginal edges of leaf primordia (Nardmann *et al.*, 2004; Cho *et al.*, 2013; Yoshikawa *et al.*, 2016), whilst in both dicots and monocots species, abundant transcripts of *WOX1/3/6* are detected in different reproductive tissues. The not-strictly-conserved expression pattern of *WOX1/3/6* implies potential functional specificities. Despite the WOX1 and WOX3/PRS redundancy in Arabidopsis, the WOX1 function in regulating medial–lateral expansion of lateral organs appears to be specific to eudicots, and this function is taken over by WOX3 homologues in monocots, further fueling the hypothesis that WOX1 function is evolutionarily dynamic. However, whether WOX1 homologues in compound leaf species outside of legumes also play a role in regulating leaf complexity is unknown.

Here, we examined the *WOX1*/*STF/LAM1* orthologous gene, *SlLAM1*, from the compound-leaf model species tomato, *Solanum lycopersicum*, and determined that it functions in controlling both leaf outgrowth and complexity. The loss-of-function mutant of *SlLAM1* displayed fewer and much smaller secondary leaflets, and also had defects in the outgrowth of the mediolateral axis of leaves and flowers, indicating that *SlLAM1* is involved in secondary leaflet initiation in the Solanaceae in addition to the conserved function in promoting lateral organ expansion. Our data shed new light on the dynamic role of WOX1 functioning and its contribution to the evolution of eudicot leaf architecture.

## Materials and methods

### Plant materials and growth conditions

Plants of tomato (*Solanum lycopersicum*, cv. Ailsa Craig) and woodland tobacco (*Nicotiana sylvestris*) were grown from seed in a greenhouse under 16/8 h light/dark conditions (150 μE m^–2^ s^–1^) at 24/20 °C with relative humidity of 50–60%. The plants were well watered and supplied with adequate nutrients.

### Vector construction, plant transformation, and genotype analyses

The full-length coding sequence of *SlLAM1* was amplified and then cloned into the pCAMBIA3301 vector to generate the *35S::SlLAM1* construct, which was transformed into *Agrobacterium tumefaciens* EHA105 strain for transformation into the *N. sylvestris lam1* mutant (Van Eck *et al.*, 2006). Two targets for CRISPR/Cas9-mediated editing of *SlLAM1* were designed by using the CRISPR-GE tool (http://skl.scau.edu.cn/) (Ma *et al.*, 2015). The gRNAs were amplified and cloned into the pYLCRISPR/Cas9P_35S_-N binary vector using the Golden Gate method (Ma *et al.*, 2015). The resulting *pYLCRISPR/Cas9P35S-N-SlLAM1* construct was used for *Agrobacterium* (EHA105 strain)-mediated transformation of tomato (Van Eck *et al.*, 2006). Genomic DNA was isolated from young leaves of T_0_ and T_1_ transgenic plants for PCR amplification, and the products were sequenced to verify the mutation status. The T_1_ plants of *CR-Sllam1-1* with genotypes that were homozygous for Target 2 (9 bp or 11 bp deletion) were chosen to perform phenotype analyses. For RNA-seq experiments, shoot apices fromT_1_ plants with mutant phenotypes (including bi-allelic ones) were collected.

### Real-time quantitative PCR

Total RNA from different tissues of the tomato and tobacco wild-types was extracted using TransZol regent (TransGen), and samples of 2 μg were reverse-transcribed using a TransScript II One-Step gDNA Removal and cDNA Synthesis SuperMix kit (TransGen) according to the manufacturer’s instructions. Real-time quantitative PCR (RT-qPCR) was performed using a TransStart Tip Green qPCR SuperMix kit (TransGen) and a LightCycler480II device (Roche). The primer sequences used are given in Supplementary Table S1. The *ACTIN* genes of tomato and tobacco were employed as the internal controls; tomato *Tubulin* and tobacco *Ubiquitin* were also used as alternative references in some experiments. Three biological replicates were performed for each sample, together with three technical replicates for each biological one. All the primers used in this study are listed in Supplementary Table S1.

### SEM analysis

Shoot apices and leaves from 1-month-old seedlings were subjected to vacuum infiltration in a fixative solution of 5% formaldehyde, 5% acetic acid, and 50% ethanol for 30 min and then kept at room temperature overnight. Before observations, the tissues were dehydrated using an ethanol series (45%, 55%, 65%, 75%, 85%, 90%, and 95%) with each step lasting for at least 1 h, and with a final step of 100% ethanol overnight. The ethanol was removed by drying in liquid CO_2_ using a Samdri critical-point dryer, and the tissues were then dissected under a stereomicroscope (SZX16, Olympus). The samples were sprayed with gold and scanned at 5 kV using an EVOLS10 device (Zeiss).

### RNA *in situ* hybridization

RNA *in situ* hybridization was performed using 8-μm sections from shoot apices of 3-week-old plants according to the method previously described by Coen *et al.* (1990). The full-length CDS of *SlLAM1* was amplified for generating DIG-labelled probes. The signals were visualized with an Olympus BX63 microscope under the DIC channel.

### Histological sectioning

Mature leaves were collected and fixed overnight in FAA solution (formalin/acetic acid/alcohol). The samples were then twice dehydrated sequentially in 50% alcohol, 100% alcohol, isopropanol, and n-butanol solutions for 4–6 h, and then submerged in glycol methacrylate resin following the protocol of Feder and O’Brien (1968). A Leica microtome was used to cut 2-μm sections, which were stained with Schiff reagent and Toluidine Blue for visualization under an Olympus BX63 microscope.

### Detection of pollen viability

Matured anthers were submerged in Alexander staining solution (Peterson *et al.*, 2010) for 30 min in darkness, and then observed under an Olympus BX63 stereomicroscope.

### Sequence alignment and phylogenetic analysis of WOXs

Amino acid sequences were downloaded for *S. lycopersicum* and *N. sylvestris* from the Sol Genomics Networks ITAG2.3 (https://solgenomics.net/) and Nsyl_ASAF01 (https://solgenomics.net/genomes/), respectively, for *Medicago truncatula* from the HAPMAP PROJECT (Mt4.0v2, http://www.medicagohapmap.org/), for *Zea mays* from MazieGDB (Zmays_493_RefGen_V4, https://maizegdb.org/), for *Oryza sativa* from the Rice Annotation Project (IRGSP-1.0, https://rapdb.dna.affrc.go.jp/index.html), for *Pisum sativum*f rom the Pea Genome project (Pisum_sativum_v1a, https://urgi.versailles.inra.fr/Species/Pisum), for *Cucurbita sativus* from the Cucumber Genome Database (Cucumber_V3, http://cucurbitgenomics.org/), for *Amborella trichopoda* from Plaza (V1.0, https://bioinformatics.psb.ugent.be/plaza/), and for *A. thaliana* from The Arabidopsis Information Resource (TAIR10, https://www.arabidopsis.org/). The amino acid sequences of the Arabidopsis WOXs were used as BLAST search queries in the genomes of the other species, with an *E*-value of<10^–5^ as the cut-off criterion. The candidate sequences were aligned using ClustalX to eliminate those without a conserved homeodomain and those that were too long, too short, or had too many gaps. A maximum-likelihood phylogenetic tree was constructed using the IQ-TREE software with a JTT+F+R6 substitution model (1000 SH-aLRT test/1000 bootstrap replications) (Nguyen *et al.*, 2015).

### RNA-seq analysis

A total of 20 shoot apex samples from 3-week-old seedlings of both the wild-type and *CR-Sllam1-1* (T_1_) plants were harvested with three biological replicates. Total RNA was extracted using TRIzol™ Reagent (Invitrogen) according to the manufacturer’s protocol. RNA quality was assessed on an Agilent 2100 Bioanalyzer. The cDNA libraries were constructed using a NEBNext® Ultra™ RNA Library Prep Kit for Illumina® (New England Biolabs) and then sequenced using an Illumina HiSeq2500 by the Gene Denovo Biotechnology Co. (Guangzhou, China). Clean reads were obtained after filtering and were mapped to the reference genome of tomato from the Tomato Sol Genomics Network (SGN) database (https://solgenomics.net/) using HISAT2, and the StringTie software was used to assemble the mapped reads and to quantify the expression of each gene as values of fragments per kilobase of transcript per million mapped reads (FPKM) (Kim *et al.*, 2015; Pertea *et al.*, 2015). Genes with *P*<0.05 and an absolute fold-change value ≥2 were considered as differentially expressed genes (DEGs),as determined using the DESeq2 software (Love *et al.*, 2014). The free online OmicShare tools (www.omicshare.com) were used for Gene Ontology (GO) analysis and examination of enrichment of Kyoto Encyclopedia of Genes and Genomes (KEGG) pathways of the DEGs, and for violin plots and sample-to-sample correlation analysis, with their default settings. The expression levels of 26 DEGs that were selected for their possible roles in leaf development were verified using RT-qPCR.

## Results

### *SlLAM1* is the ortholog of *STF* and *LAM1*

**Fig. 1.**
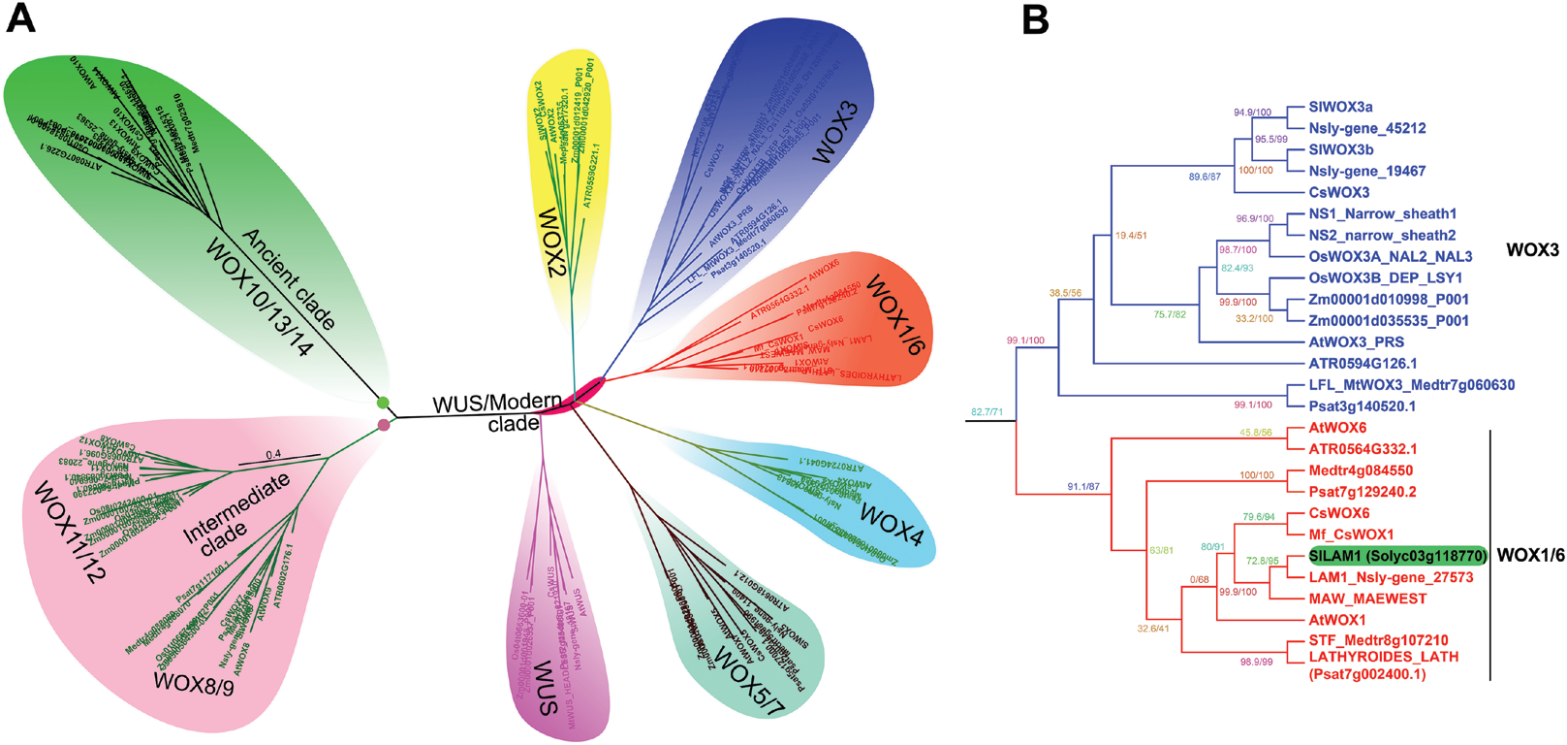
Phylogenetic relationships of WOX family members. (A) Protein sequences of WOX were collected for phylogenetic analysis from Arabidopsis (15), Medicago (15), tomato (10), cucumber (11), tobacco (10), rice (11), maize (18), pea (15), *Amborella trichopoda* (9), and petunia (1). The WOX members were divided into three clades: Ancient, Intermediate, and WUS/Modern. See Supplementary Fig. S1 for detail. (B) Detailed view of the WOX1/6 and WOX3 subclades. SlLAM1 (Solyc03g11870) is closely related to LAM1 and MAW from the Solanaceae and to *Medicago* STF. The results of 1000 SH-aLRT tests (%) and 1000 bootstrap replications (%) are indicated. Detailed information for the WOX transcription factors in tomato is provided in Supplementary Table S2.

There are 15 *WOX* genes in Arabidopsis and several of them have been reported to play important roles in leaf and floral organ development, embryogenesis, and stem cell maintenance in shoot and root apical meristems (Laux *et al.*, 1996; Haecker *et al.*, 2004). Out of these 15 genes, *WOX1* and *WOX3/PRS* redundantly control leaf-blade outgrowth (Vandenbussche *et al.*, 2009; Nakata *et al.*, 2012), and PRS is reported to function in the recruitment of leaf primordial founder initials from the SAM (Nardmann *et al.*, 2004). If this assumption is true, *WOX1* and *PRS* may also be expected to be involved in the control of leaflet numbers in compound leaf species. However, in *M. truncatula*, a legume species with compound leaves, leaf complexity appears to be unaffected in the *stf* loss-of-function mutant, but *STF/MtWOX1* and *LFL/MtWOX3* function in separate pathways (Niu*et al*., 2015). To gain better insights about the independent functions of WOX1 homologues in leaf-blade outgrowth and possible contributions to leaf complexity, we studied the tomato WOX1 homologue SlLAM1. With its sympodial stem growth habit and cymose inflorescences, tomato is a compound leaf species in the Solanaceae and is an excellent model to study leaf, inflorescence, and fruit development, in which the functions of WOX genes are likely to be apparent. To verify the WOX proteins in tomato, we used the sequences of Arabidopsis WOXs to conduct a BLAST search of the tomato genome using an *E*-value of <10^–5^ as the cut-off criterion, and this identified 10 typical WOX proteins (Supplementary Table S2). We also probed the *M. truncatula*, pea, *C. sativus*, *N. Sylvestris*, rice, and maize genomes, in which mutants of *WOX1* or *WOX3* have been reported, and identified a total of 106 *WOX* genes, representing species from the Fabaceae, Cucurbitaceae, Poaceae, and Solanaceae. We then conducted a phylogenetic analysis of the corresponding WOX proteins, which included 15 from Arabidopsis, 11 from *C. sativus*, 15 from *M. truncatula*, 15 from pea, 11 from rice, 18 from maize, 10 from tomato, 10 from *N. sylvestris*, and one from petunia (MAW), using the IQ-TREE software (Nguyen *et al.*, 2015). This analysis indicated that whilst the ancient clade was represented by just one subclade (WOX10/13/14), the intermediate clade was divided into two subclades (WOX8/9 and WOX11/12), and the WUS/Modern clade into six subclades (WUS, WOX1/6, WOX2, WOX3, WOX4, and WOX5/7) (Fig. 1A, Supplementary Fig. S1), consistent with the phylogeny of the WOX transcription factor family in plants (Wu *et al.*, 2019). The 10 SlWOX members could also be grouped into three clades: the WUS/modern clade (7), the intermediate clade (2), and the ancient clade (1), consistent with the phylogeny of Arabidopsis WOX proteins (van der Graaff *et al.*, 2009). The WOX3 subclade was represented by two members and each of the other eight subclades was represented by a single SlWOX (Fig. 1B, Supplementary Fig. S1). Based on this comprehensive phylogenetic analysis, we identified SlLAM1 (Solyc03g118770) in tomato as being a single-copy gene closely related to LAM1, MAW, and STF (Fig. 1B).

### *SlLAM1* is strongly expressed in leaves, fruit, and flowers

**Fig. 2.**
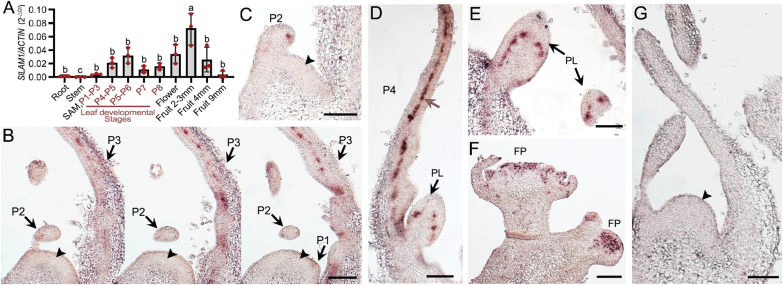
Expression patterns of *SlLAM1* in vegetative and reproductive organs in tomato. (A) Expression levels of *SlLAM1* in different organs as determined by RT-qPCR. Expression is relative to that of *ACTIN*, which was used as the internal control. The developmental stages of the primordia (P1–P8) are as defined by (Kang and Sinha, 2010). *SlLAM1* is widely expressed, with relatively high levels of transcripts detected in P4–P6 leaf primordia, flowers, and young fruit. Individual data points are shown together with the means (±SD), *n*=3. Different letters indicate significant differences as determined using ANOVA followed by Tukey’s test (*P*<0.05). (B–F) RNA *in situ* hybridization analysis of *SlLAM1* expression in the primordia at (B) the P1–P3 stages (serial sections), (C) the P2 stage, and (D) the P4 stage, and in (E) the primary leaflets (PL) and (F) the flower primordium (FP). (G) The sense probe was used as negative control. *SlLAM1* is specifically expressed in the mediolateral axis of the lamina but not in the shoot apical meristems (arrowheads), and high expression levels are also detected in the flower primordium. The brown arrow in (D) denotes the adaxial–abaxial boundary layer. Scale bars are 100 μm.

To determine the function of *SlLAM1*, we first examined its expression pattern using real-time quantitative PCR (RT-qPCR). *SlLAM1* was expressed widely in the vegetative and reproductive organs, with relatively high levels of transcript accumulation detected in leaf primordia at the P4–P6 stages, in flowers, and in young fruit (Fig. 2A). To examine expression patterns in the early stages of leaf and flower development in more detail, RNA *in situ* hybridization assays were used. Transcripts of *SlLAM1* were found at relatively low levels in leaflet primordia at the P1–P3 stages, but not in the SAM (Fig. 2B–G).Subsequently, enrichment was observed in the adaxial–abaxial boundary layer of the leaf (P4), and primary leaflet primordia (Fig. 2D, E) and floral organ primordia (Fig. 2F), similar to that of *STF* in *M. truncatula* (Tadege *et al.*, 2011; Zhang *et al.*, 2014). These results suggested that *SlLAM1* may function in leaf and flower development, consistent with WOX1 functioning in other eudicots.

### Overexpression of *SlLAM1* can rescue the leaf phenotype of the tobacco *lam1* mutant

**Fig. 3.**
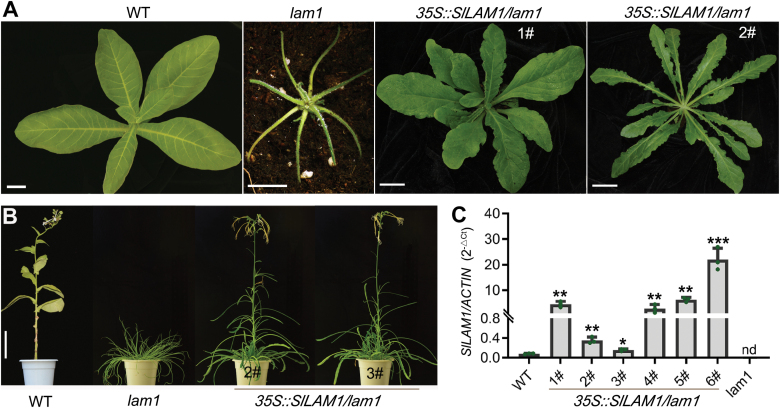
Complementation of the tobacco *lam1* mutant by tomato *SlLAM1*. (A) Phenotypes of 8-week-old plants of the *N. sylvestris* wild-type (WT), the *lam1* mutant, and two transgenic *35S::SlLAM1/lam1* lines. (B) The non-flowering phenotype of *lam1* is rescued in *35S::SlLAM1/lam1* transgenic lines. Plants at 4 months old are shown. Scale bars in (A, B) are 2 cm. (C) Expression levels of *SlLAM1* in the WT, *lam1* mutant, and transgenic lines. Expression is relative to that of *ACTIN*, which was used as the internal control. Data are means (±SD), *n*=3. Significant differences compared with the WT were determined using unpaired two-sample *t*-tests: *P<0.05, **P<0.01, ***P<0.001; nd, not detected.

To further investigate the function of *SlLAM1* in Solanaceae, we conducted a complementation test by generating a *35S::SlLAM1* construct and transforming it into the *lam1* mutant of *N. sylvestris* (woodland tobacco). We obtained six transgenic lines that showed various degrees of complementation in leaf-blade expansion (Fig. 3A, Supplementary Fig. S2). The *lam1* mutant has a very short stem and is non-flowering, whereas two of the *35S::SlLAM1/lam1* transgenic plants had a wild-type-like stem and were able to flower under standard growth conditions (Fig. 3B).The other four transgenic plants did not show rescue of the non-flowering phenotype, and this might have been due to the higher accumulation of *SlLAM1* transcripts in these lines (Fig. 3C, Supplementary Fig. S2). However, the *35S::SlLAM1/lam1* plants were still sterile, probably due to only partial complementation (Supplementary Fig. S3). These results suggested that the protein features of SlLAM1 responsible for leaf expansion are conserved in tomato and tobacco; however, the ectopic expression assays did not enable us to determine whether differential effects existed in reproductive organ growth and flowering. Transgenic plants with *SlLAM1* driven by the native or *LAM1* promoter would be useful for obtaining information in this regard.

### SlLAM1 regulates compound leaf development in tomato

**Fig. 4.**
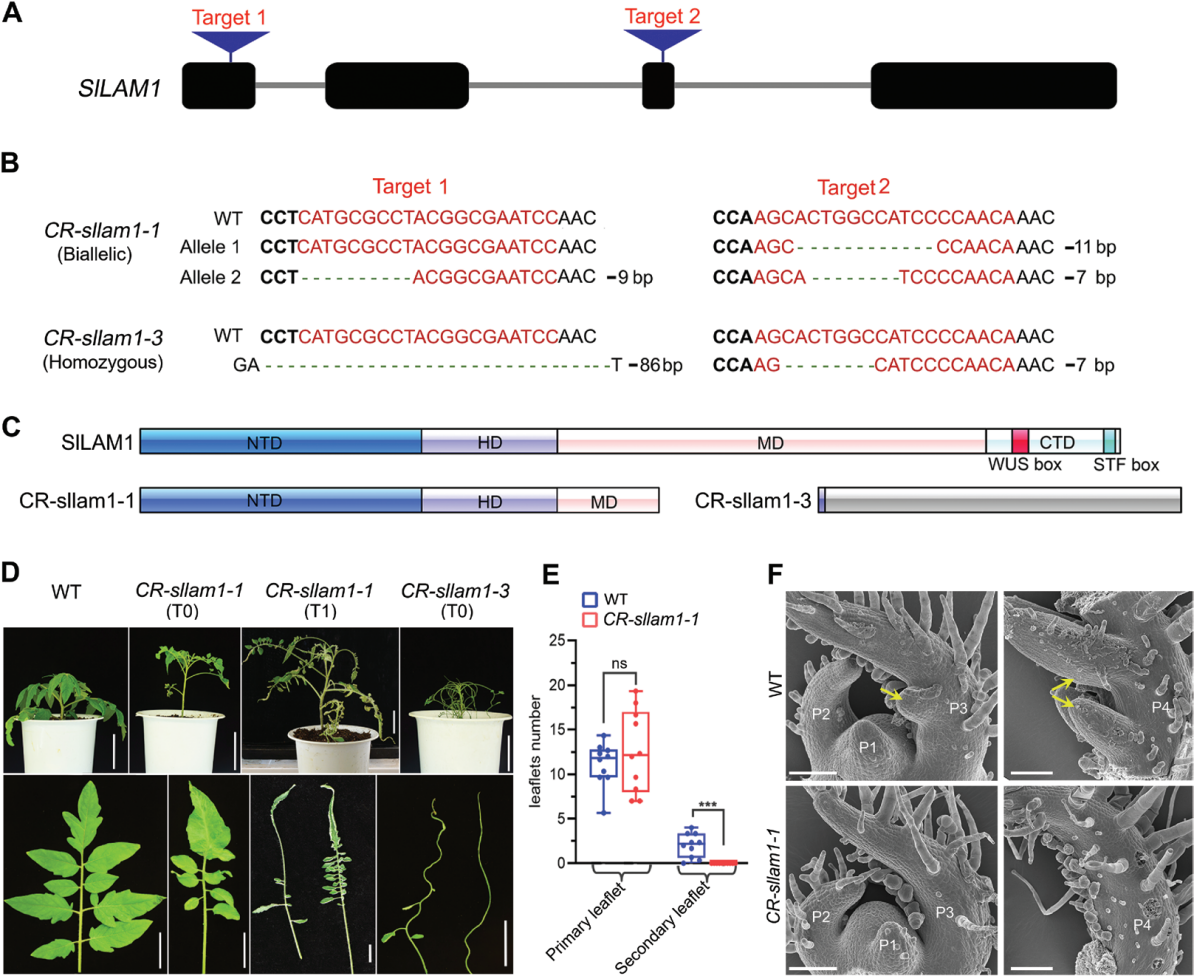
Mutation sites of CRISPR/Cas9-edited tomato *Sllam1* mutants and resulting leaf phenotypes. (A) Schematic diagram of the designed target sites in the *WOX1* genomic sequence. The black boxes indicate exons and the gray lines indicate introns. (B) Mutation status of the *CR*-*lam1* mutants. The T_0_ plant of *CR-Sllam1-1* is a bi-allelic mutant, while *CR-Sllam1-3* is homozygous at both target sites. The bold letters in black are the protospacer adjacent motif sequences, red letters indicate the target sequences, and the dashes denote deleted nucleobases. (C) Sllam1 proteins in the CRISPR/Cas9-edited *Sllam1* mutants (T_0_). The schematic diagram shows the wild-type (WT) SlLAM1 protein containing the N-terminal domain (NTD), homeodomain (HD), middle domain (MD), C-terminal domain (CTD), the WUS box (red), and the STF box (cyan). In the *CR-Sllam1-1* plant, parts of the MD and the CTD were deleted, while miscoded Sllam1 protein starting from the NTD (gray box) occurred in the *CR-Sllam1-3* mutant. (D) Phenotypes of 8-week-old plants of the WT, *CR-Sllam1-1* mutant (T_0_, T_1_), and *CR-sllam1-3* (T_0_). Scale bars are 2 cm. The *CR-Sllam1-1* (T_1_) mutants all displayed the same defective phenotypes and no WT-like plants were found in these lines. Compared with the WT, *CR-Sllam1-1* and *CR-Sllam1-3* have narrower lamina and under-developed secondary leaflets. (E) Boxplot of primary and secondary leaflet numbers for the WT and *CR-Sllam1-1* (T_1_) mutants. Measurements were taken on plants at 2 months old, and three leaves (5th–7th) from 10 individual plants were measured. Significant differences were determined using unpaired two sample *t*-tests: ***P<0.001; ns, not significant. (F) SEM images of shoot apical morphology in the WT and *CR-Sllam1-1* (T_1_) mutant. Scale bars are 100 μm. No lateral leaflet primordia were initiated at P3 or later stages in *CR-Sllam1-1*.

To better understand the function of *WOX1* in tomato, we generated loss-of-function mutants of *SlLAM1* using CRISPR/Cas9-mediated genome editing (Ma *et al.*, 2015). Two targets were selected and integrated to one construct, with the aim of enhancing the editing efficiency (Fig. 4A). The two targets were located in the first and the third exon of the CDS sequence of *SlLAM1*. We acquired two representative mutant lines and confirmed their mutation sites by PCR amplification and sequencing (Fig. 4B). The *CR-Sllam1-1* (T_0_) line was a bi-allelic mutant with a 9-bp deletion (in-frame) in target 1, and 11-bp and 7-bp deletions in target 2 (Fig. 4B). The *CR-Sllam1-3* (T_0_) line was homozygous with a 86-bp deletion near target 1 and a 7-bp deletion in target 2 (Fig. 4B). Compared with the amino acid sequences of STF, LAM1, and wild-type SlLAM1, the C-terminal domain and part of the middle domain of SlLAM1 were deleted in the *CR-Sllam1-1* mutant, while a large fragment frame shift occurred in the *CR-Sllam1-3* mutant (Fig. 4C, Supplementary Fig. S4).

The two independent CRISPR-generated mutant alleles displayed similar phenotypes, suggesting that this was indeed caused by the loss-of-function of *SlLAM1* (Fig. 4D). In the *CR-Sllam1-1* (T_0_) line, the phenotype was mild and the mediolateral axis growth of the leaf was not distinctly altered, but the leaf margin was slightly serrated and leaflets were shorter than that of the wild-type (WT). In contrast, the *CR-Sllam1-1* (T_1_) and *CR-Sllam1-3* (T_0_) lines displayed strong defects in leaf growth in the mediolateral axis (Fig. 4D). These *CR* mutants had narrower terminal and lateral leaflets, and in some cases the blades developed into vestigial strip-like structures, similar to that of the tobacco *lam1* mutant (McHale, 1992). This narrow-leaflet phenotype of *CR-Sllam1* indicated a conserved function of *SlLAM1* in governing blade expansion, similar to previous reports for its orthologous genes(McHale, 1992; Vandenbussche *et al.*, 2009; Tadege *et al.*, 2011; Zhuang *et al.*, 2012). In addition, *CR-Sllam1* exhibited alterations in leaflet number and length (Fig.4D). The number was variable in a single mutant; compared with the WT, many of the early leaves displayed fewer or hardly any primary leaflets, while some later leaves had more leaflets but secondary leaflets had almost vanished. We quantified the number of leaflets in compound leaves of the *CR-Sllam1-1* (T_1_) mutant and found that there were no obvious differences for the primary leaves, but the number of secondary leaflets was significantly reduced (Fig. 4E). We also used SEM to examine shoot apical tissue from 1-month-old plants and found that the primary lateral leaflet primordia were initiated at the P3 stage in the WT, but no primordia were present in the *CR-Sllam1-1* (T_1_) mutant at either the P3 or P4 stages (Fig. 4F). These results suggested that the loss-of-function of *SlLAM1* might affect the initiation of secondary leaflets in tomato, in addition to its conserved function in blade expansion.

**Fig. 5.**
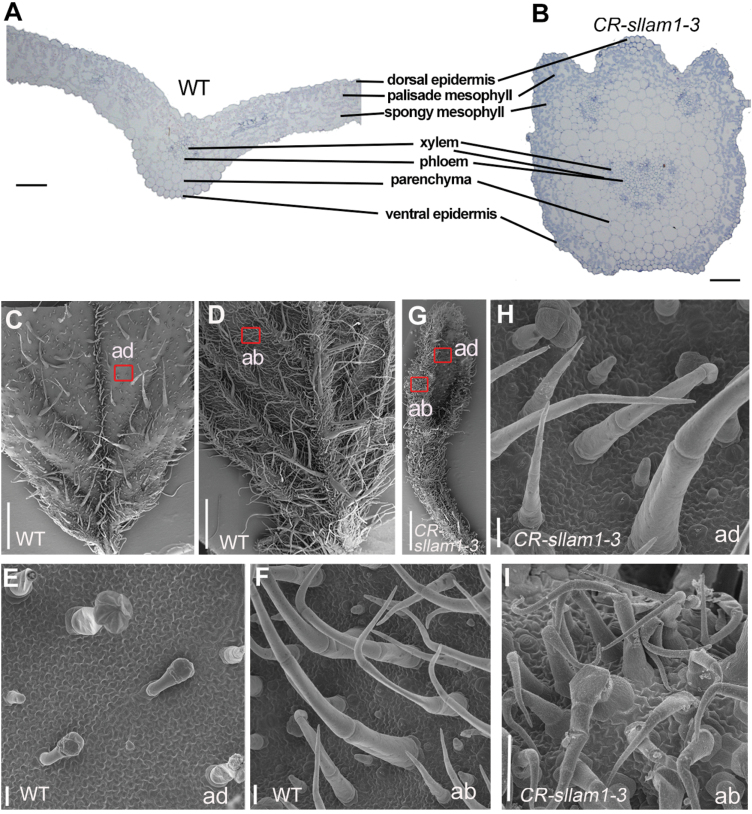
Adaxial/abaxial patterning in the tomato wild-type and the CRISPR/Cas9-edited *CR-Sllam1-3* mutant. (A, B) Transverse sections of matured lateral leaflets of (A) the wild-type (WT) and (B) the *CR-Sllam1-3* mutant. The leaflet blade is not expanded in the mutant, but the layers of leaf tissues are similar to those of the WT. (C–I) SEM images of adaxial and abaxial surfaces of (C–F) the WT and (G–I) the mutant. (C) Adaxial (ad) and (D) abaxial (ab) surface of young laminae. Magnified images of the regions in the red boxes are shown in (E) and (F), respectively. (G) Young leaflet lamina of the mutant is shown, and magnified images of the regions in the red boxes are shown in (H) and (I). Scale bars are 100 μm in (A, B), 1 mm in (C, D, G), and 20 μm in (E, F, H, I).

Since the *Sllam1* mutants sometimes showed a strip-like leaf, we used semi-thin sections to examine the morphological alterations in the severely defective leaves of *CR-Sllam1-3* (T_0_) to see how the growth of the adaxial/abaxial surfaces were affected. In the WT, the lamina extended from the midrib to the blade margin with a distinct arrangement of tissue layers (Fig. 5A). The arrangement of the layers was similar in the *CR-Sllam1-3* mutant but the leaf blade was not extended (Fig. 5B). Examination using SEM showed that although there were more trichomes on the abaxial leaf surface of the *CR-Sllam1-3* mutant, the epidermal cells of the WT and the mutant were similar on both the adaxial and abaxial surfaces (Fig. 5C–I, Supplementary Fig. S5), suggesting that the adaxial/abaxial identity was not significantly affected. These results therefore indicated that SlLAM1 regulates tomato leaf development mainly by governing the mediolateral axis growth of the blade.

### SlLAM1 regulates reproductive organ development in tomato

**Fig. 6.**
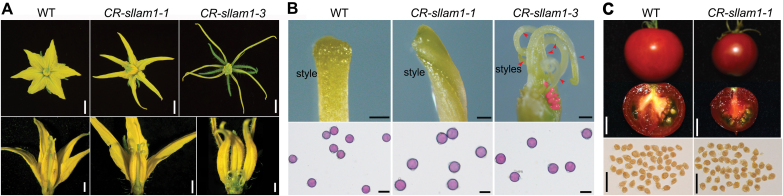
Floral organs and fruit phenotypes of the tomato wild-type and CRISPR-edited *CR-Sllam1* mutant. (A) Phenotypes of floral organs in the wild-type (WT) and two mutant lines. Narrower and unfused petals occur in the mutants. (B) Multiple or unfused styles in the *CR-sllam1* mutants (upper panels). The arrowheads indicate the multiple styles in *CR-sllam1-3* (T_0_), and red shading indicates the exposed ovules that result from the unfused carpels. The viability of pollen grains is not significantly changed in the mutants, as indicated by Alexander staining (lower panels). (C) The fruits but not the seeds of the *CR-sllam1-1* (T_0_) mutant are smaller than those of the WT. Ripe fruits and seeds are shown. Scale bars are 2 cm in (A, C), 100 μm in (B, upper), and 10 μm in (B, lower).

The *CR-Sllam1* mutants also showed significant variation in their floral organs. The WT had fused carpels and six petals fused at the base of the corolla (Fig. 6A), whilst in the *CR-Sllam1-1* mutant the petals were narrower and separated from each other, and the style was slightly twisted (Fig. 6B). In the *CR-Sllam1-3* (T_0_) mutant, the petals appeared choripetalous and acicular, the sepals were narrower than those of the WT and *CR-Sllam1-1* mutant, and the carpels were dehiscent with multiple slender styles pointing down (Fig. 6B). Consistent with the strength of the mutant phenotypes, we observed that *CR-Sllam1-1* (T_0_) was fertile, while the single *CR-Sllam1-3* (T_0_) plant and the *CR-Sllam1-1* (T_1_) plants were sterile and no seeds could be obtained from them. We tested the viability of mature pollen using Alexander staining and found that it was not significantly affected (Fig. 6B), indicating that the defects in the gynoecium were the cause of the sterility phenotype. Interestingly, we found that *CR-Sllam1-1* (T_0_) plants produced smaller fruit compared to WT plants (Fig. 6C) but that there was no difference in seed size (Fig. 6C), indicating a critical role for SlLAM1 in fruit development in which the bi-allelic mutant leads to reduced fruit size and the homozygous mutant leads to complete sterility.

### Differential gene expression between *CR-Sllam1-1* and the WT

**Fig. 7.**
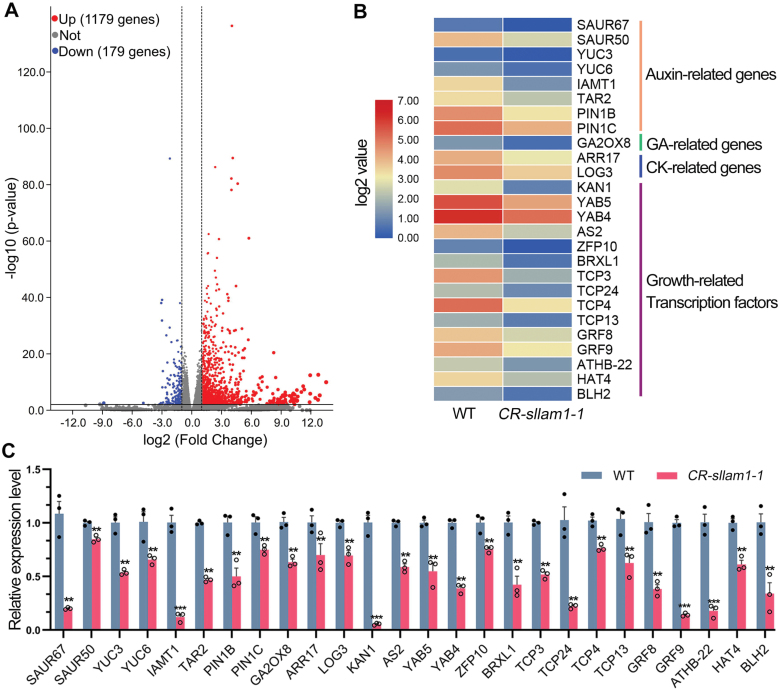
Differentially expressed genes between the tomato wild-type and the *CR-sllam1-1* mutant (T_1_). (A) Volcano plot showing the significantly differentially expressed genes (DEGs). (B) Heatmap showing 26 DEGs related to phytohormones and transcription factors. Mean FPKM values were used to construct the heatmap. (C) Confirmation of the relative expression levels of the DEGs between *CR-sllam1* and the WT as determined by RT-qPCR. Expression is relative to that of *ACTIN*, which was used as the internal control. Individual data points are shown together with the means (±SD), *n*=3. Significant differences compared with the WT were determined using unpaired two-sample *t*-tests: **P<0.01, ***P<0.001.

To gain insights into the potential regulatory pathway of *SlLAM1*, we conducted RNA-seq using tissue from the shoot apex of 3-week-old seedlings of the *CR-Sllam1-1* mutant (T_1_) and the WT (Supplementary Table S3). The RNA-seq showed high reproducibility (Supplementary Fig. S6) and we identified 1358 differently expressed genes (DEGs; adjusted *P*-value <0.05) in the mutant, of which 1179 were up-regulated and 179 were down-regulated (Fig. 7A, Supplementary Tables S4, S5). Genes with negative regulatory functions were significantly enriched (Supplementary Fig. S7), which is consistent with the primarily repressive function of WOX1 homologues (Lin *et al.*, 2013; Zhang *et al.*, 2014). Analysis of KEGG pathways showed that genes belonging to ‘plant hormone signal transduction’ (ko04075) were enriched (Supplementary Fig. S8), and such genes have been widely reported to be involved in leaf development (Bar and Ori, 2014). Interestingly, the auxin biosynthetic and metabolic genes *YUCCA3* and *IAMT1*, the auxin transport component gene *PIN*, and the auxin-responsive gene *SAUR67* were all highly down-regulated (Fig. 7B, C, Supplementary Fig. S9, Supplementary Table S6), suggesting that SlLAM1 may regulate leaflet development via this hormone. Expression of several transcription factors related to leaf polarity and leaf growth were also down-regulated (Fig. 7B, C), indicating that *SlLAM1* might recruit multiple genes in the process of regulating leaf development in tomato.

## Discussion

*WOX1* genes are reported to be involved in lateral organ growth and development in several species. Loss-of-function mutants usually display abnormal leaf and flower phenotypes, for example *lam1* in *N. sylvestris*, *maw* in petunia, *stf* in *M. truncatula*, *lath* in pea, and *mf* in cucumber (Vandenbussche *et al.*, 2009; Tadege *et al.*, 2011; Nakata *et al.*, 2012; Zhuang *et al.*, 2012; Costanzo *et al.*, 2014; Niu*et al*., 2018; Wang *et al.*, 2020). Here, we characterized the *WOX1* homologous gene *SlLAM1* in tomato, and identified both novel and conserved aspects of WOX1 regulatory roles in plant growth and development. Our results demonstrated that SlLAM1 affected the leaflet number of compound leaves in addition to its commonly described functions in controlling lateral expansion of the leaf blade and floral organs (Figs 3, 4). It appeared that the number of primary leaflets was reduced in the early leaves of the CRISPR/Cas-9-edited line C*R-Sllam1-1* but not in leaves that were produced later, suggesting that leaflet initiation may have been delayed. This has recently been confirmed by an independent study of *SlLAM1* by Du *et al.* (2020), who carried out detailed quantification of leaflet numbers and examined the expression patterns of the *DR5::VENUS* auxin-response reporter and found that leaflet initiation is indeed delayed in *Sllam1-1*. Since fewer secondary leaflets were found in both *CR-Sllam1* (Fig. 4) and the *Sllam1-1* mutants (Du *et al.*, 2020), it is reasonable to assume that SlLAM1 affects their initiation. We also found that knocking down *SlLAM1* led to narrow leaflets in the *CR-Sllam1-1* line in which the C-terminal and part of the middle domain were deleted, whilst the *CR*-*Sllam1-3* line in which almost all the domains were deleted showed the most severe leaf defects. This difference might further indicate that functioning of the homeodomain and C-terminal domain are essential for leaflet outgrowth, which is consistent with previous results (Zhang *et al.*, 2014).

Interestingly, our RNA-seq analysis highlighted the expression of auxin biosynthesis, auxin transport, and auxin-response genes as all being highly enriched in the *CR-Sllam1-1* mutant compared with the WT (Fig. 7), and this phytohormone is known to be involved in a wide range of plant developmental processes. Similar enrichment in auxin-related gene expression has also consistently been identified in the *mf* mutant in cucumber (Niu *et al.*, 2018), the *stf* mutant in *M. truncatula* (Tadege *et al.*, 2011), and in transgenic plants with induced WOX1 expression in Arabidopsis (Nakata *et al.*, 2018), suggesting that *SlLAM1* may indeed interact with several auxin response and signaling components during leaflet initiation, leaf-blade expansion, and floral organ development. In tomato, LYRATE (LYR) is indispensable for regulating the auxin response during the initiation of leaflet primordia (David-Schwartz *et al.*, 2009), and the auxin-response inhibitor ENTIRE (E, SIIAA9) and the CUC transcription factor GOBLET (GOB) can integrate with auxin to regulate leaflet initiation and serration (Ben-Gera *et al.*, 2012). Specific interactions between SlLAM1 and LYR, CUC, or GOB are yet to be established. Notably, a recent report has shown that *SlLAM1* functions downstream of *E* in mediating leaflet initiation and blade expansion (Du *et al.*, 2020). The *e* mutation, which increases the auxin response, slightly rescues the blade width of *Sllam1-1*, indicating that there might be additional genes acting downstream of auxin signaling to promote leaf-blade expansion (Du *et al.*, 2020). This inference is partially supported by our results that showed that several auxin-responsive *SAUR* genes and auxin-related transciption factors were down-regulated in *CR-Sllam1-1*, including members of the *TEOSINTEBRANCHED1/CYCLOIDEA/PROLIFERATION CELL FACTOR* (*TCP*) and *GROWTH-REGULATING FACTOR* (*GRF*) famlies (Fig. 7). Further research is required to determine whether these auxin-related genes are indispensable for *SlLAM1*-mediated leaf developemnt.

*Medicago* STF has been shown to promote cytokinin activity in transgenic switchgrass by repressing cytokinin-degrading ezymes (Wang *et al.*, 2017), and a direct connection between WUS and cytokinin has been demonstrated in Arabidopsis (Leibfried *et al.*, 2005; Meng *et al.*, 2017; Wang *et al.*, 2017; Zhang *et al.*, 2017; Zubo *et al.*, 2017; Ito *et al.*, 2018). In addition, expression of *WOX1* and *WOX3* in apple is strongly up-regulated in response to cytokinin treatment (Li *et al.*, 2019b), and auxin can also induce the expression of *CsWOX1b* and *CsWOX3* in cumcuber (Gu *et al.*, 2020). In Arabidopsis, expression of *WOX1* and *PRS* can be directly up-regulated by the auxin signaling component Auxin Response Factor 5 (ARF5), but it is suppressed by ARF2, ARF3, and ARF4 (Guan *et al.*, 2017). These studies together with the genetic interactions between auxin-related genes and *SlLAM1* determined by Du *et al.* (2020) imply that both auxin and cytokinin are interconnected with *WOX1*, and that they might be in some form of regulatory feedback loop. Hence, future studies need to focus on auxin- and cytokinin-mediated pathways and their cross-talk in order to shed light on the multiple functions of SlLAM1 and to comprehensively understand the genetic networks involved in leaf and flower developmental.

In the petunia *maw* mutant, the petals and carpels are unfused and female fertility is reduced (Vandenbussche *et al.*, 2009; Segatto *et al.*, 2013). Similarly, in the *stf* mutant of *M. truncatula*, the petals are unfused and the carpel is open with protruding ovules, which results in complete female sterility (Tadege *et al.*, 2011). The strong alleles in pea *lath* mutants also result in female sterility (Zhuang *et al.*, 2012). In the Arabidopsis *wox1 prs* double-mutant, the carpels are fused, but fertility appears to be unaffected despite narrow and unfused petals (Vandenbussche *et al.*, 2009). In the cucumber *mf* mutant, on the other hand, both male and female sterility are observed, with rare fertility under field conditions producing shorter seeds and fruits (Niu *et al.*, 2018). The *N. sylvestris lam1* mutant is completely blocked in stem elongation, but when bolting is induced with high temperature, characteristic unfused petals and carpels are observed together with sterility phenotypes (McHale, 1992; Tadege *et al.*, 2011). These results indicate that the narrow leaf and petal phenotypes of *wox1* loss-of-function mutants are common and they show a conserved role for WOX1 in lateral organ expansion; however, the defects in carpel development are variable and lead to phenotypes that range from reduced fertility to sterility depending on species. Compared with these *wox1* mutants in other species, the tomato *CR-Sllam1* mutants showed similar defects in the form of narrow leaves, unfused petals, and unfused and sterile carpels (Figs 4, 6), but they also showed additional effects in the development of the compound leaves and in the regulation of fruit size. The significant reduction in secondary leaflets in *CR-Sllam1* (Fig. 4D–F) reveals that *SlLAM1* plays a novel role in regulating their initiation in tomato that is not apparent in legumes. In addition, the significantly reduced fruit size in the heterozygotes (Fig. 6C) suggests that SlLAM1 is involved not only in the regulation of fertility but also in the regulation of embryo development that determines final fruit and seed size. This is consistent with the observation in cucumber that the rarely fertile *mf* mutants produce shorter fruits and abnormal seeds (Niu *et al.*, 2018); the female-sterility phenotype might have precluded identification of this defect in other species. In agreement with this, the LATH in pea has been found to physically interact with the regulatory proteins controlling organ size BIGGER ORGANS (BIO) and ELEPHANT EAR-LIKE LEAF1 (ELE1), which target*PsGRF5* (Li *et al.*, 2019a). A re-evaluation of the *wox1* mutation in these species with conditional WOX1 activity might help to further uncover its role in the regulation of seed size and embryogenesis.

The potential involvement of WOX1 in the development of male reproductive organs has been proposed in cucumber, in which CsWOX1physically interacts with NOZZLE/SPOROCYTELESS (NZZ/SPL), a protein required for cell division and differentiation of the anther cell wall, to regulate sporogenesis (Niu *et al.*, 2018). RNA-seq analysis also revealed that homologues of genes known to be important for tapetal and microspore development are down-regulated in the cucumber *mf* mutant, such as *CsSPL*, *CsDYT1*, *CsMS1*, *CsAMS*, *CsGAMYB*, and *CsMYB103* (Niu*et al*., 2018), suggesting that CsWOX1 plays a role in male reproductive organ development by directly or indirectly promoting the expression of genes involved in sporogenesis. Previous studies have demonstrated that *Medicago* STF physically interacts with MtTPL and acts as a transcriptional repressor in promoting the expansion of the leaf blade and floral organs (Lin *et al.*, 2013; Zhang *et al.*, 2014). A similar repressive activity of WUS has also been shown to mediate its functioning in stem cell maintenance in vegetative and reproductive meristems in Arabidopsis (Dolzblasz *et al.*, 2016), even though WUS had previously been reported to be a bifunctional transcription factor that acts as a repressor in vegetative SAMs and as an activator in reproductive organ development (Ikeda *et al.*, 2009). *LOOSE FLOWER* (*LFL*), the *M. truncatula WOX3* gene, has also been identified to function as a repressor in regulating floral organ development (Niu *et al.*, 2015). Therefore, it is likely that SlLAM1 also acts as a transcriptional repressor in regulating leaf-blade outgrowth, leaflet number, petal expansion, and carpel development. Identifying the key targets required for the accomplishment of each of these processes will further our understanding of the molecular mechanisms by which SlLAM1 orchestrates these multiple functions.

In summary, our study has uncovered the involvement of SlLAM1 in the regulation of secondary leaflet initiation and possibly fruit size in tomato, in addition to regulating leaf-blade expansion and floral organ development. We have thus expanded the range of developmental processes regulated by *WOX1* genes in plants, paving the way for further improving our understanding of the evolution of complex lateral organs.

## Supplementary data

The following supplementary data are available at *JXB* online.

Table S1. List of primers used in this study.

Table S2. Details of the 10 WOX proteins identified in tomato.

Table S3. RNA-seq statistics for the wild-type and *CR-Sllam1* samples.

Table S4. Significantly up-regulated genes in *CR-Sllam1* relative to the wild-type.

Table S5. Significantly down-regulated genes in *CR-Sllam1* relative to the wild-type.

Table S6. FPKM values of selected genes that were significantly down-regulated in *CR-Sllam1-1*.

Fig. S1. Phylogenetic relationships of WOX proteins among different species.

Fig. S2. Complementation of the tobacco *lam1*phenotype by *SlLAM1*.

Fig. S3. Sterility phenotype of transgenic *35S::SlLAM1/lam1* plants.

Fig. S4. Multiple sequence alignments of STF, LAM1, SlLAM1, and the two *CR-Sllam1* mutant proteins.

Fig. S5. SEM images of the adaxial and abaxial leaf surfaces of the wild-type and the *CR-Sllam1-1* (T_1_) mutant.

Fig. S6. Evaluation of the reproducibility of the RNA-seq experiment.

Fig. S7. GO enrichment plot of differentially expressed genes between the *CR-Sllam1-1* mutant and the wild-type.

Fig. S8. KEGG enrichment plot of differentially expressed genes between the *CR-Sllam1-1* mutant and the wild-type.

Fig. S9. RT-qPCR results obtained using alternative reference genes.

eraa574_suppl_Supplementary_File001

eraa574_suppl_Supplementary_File002

## Data Availability

The raw data of the RNA-seq experiment have been deposited in the NCBI Sequence Read Archive (https://www.ncbi.nlm.nih.gov/sra) under BioProject PRJNA660202. All other data supporting the findings of this study are available within the paper and within its supplementary materials published online.
