# Supplementary material for: The WOX family transcriptional regulator SlLAM1 controls compound leaf and floral organ development in Solanum lycopersicum
Source: J Exp Bot. 2020 Dec 5;72(5):1822–35. doi: 10.1093/jxb/eraa574 (PMC7921304; doi:10.1093/jxb/eraa574)
Supplement: eraa574_suppl_Supplementary_File002 [file eraa574_suppl_supplementary_file002.pdf]

**Supplementary data for**

# The WOX family transcriptional regulator SlLAM1 controls compound leaf and floral organ development in *Solanum lycopersicum*

Chaoqun Wang[†], Baolin Zhao[†], Liangliang He, Shaoli Zhou, Ye Liu, Weiyue Zhao, Shiqi Guo, Ruoruo Wang, Quanzi Bai, Youhan Li, Dongfa Wang, Qing Wu, Yuanfan Yang, Yu Liu, Million Tadege [*], Jianghua Chen[*]

**Content**

Table S1. List of primers.

Table S2. Features of the WOXs proteins in tomato.

Table S3. RNA-Seq statistics of WT and *CR-sllam1* samples.

Table S4. Significantly up-regulated genes in *CR-sllam1.*

Table S5. Significantly down-regulated genes in *CR-sllam1.*

Table S6. FPKM value of chosen significantly down-regulated genes in *CR-sllam1-1.*

Fig. S1. Details phylogenetic relationships of the WOX.

Fig. S2. Complementation of *lam1* phenotype by *SlLAM1.*

Fig. S3. Sterility phenotype of *35S::SlLAM1/lam1* plant.

Fig. S4. Multiple Sequences alignment of STF, LAM1, SlLAM1, and the two CR-sllam1 mutant proteins.

Fig. S5. Adaxial/abaxial patterning in *CR-sllam1-1* (T1) mutant.

Fig. S6. Evaluation of reproducibility of RNA-seq experiment.

Fig. S7. GO enrichment plot of DEGs.

Fig. S8. KEGG enrichment plot of DEGs.

Fig. S9. RT-qPCR results using alternative reference genes.

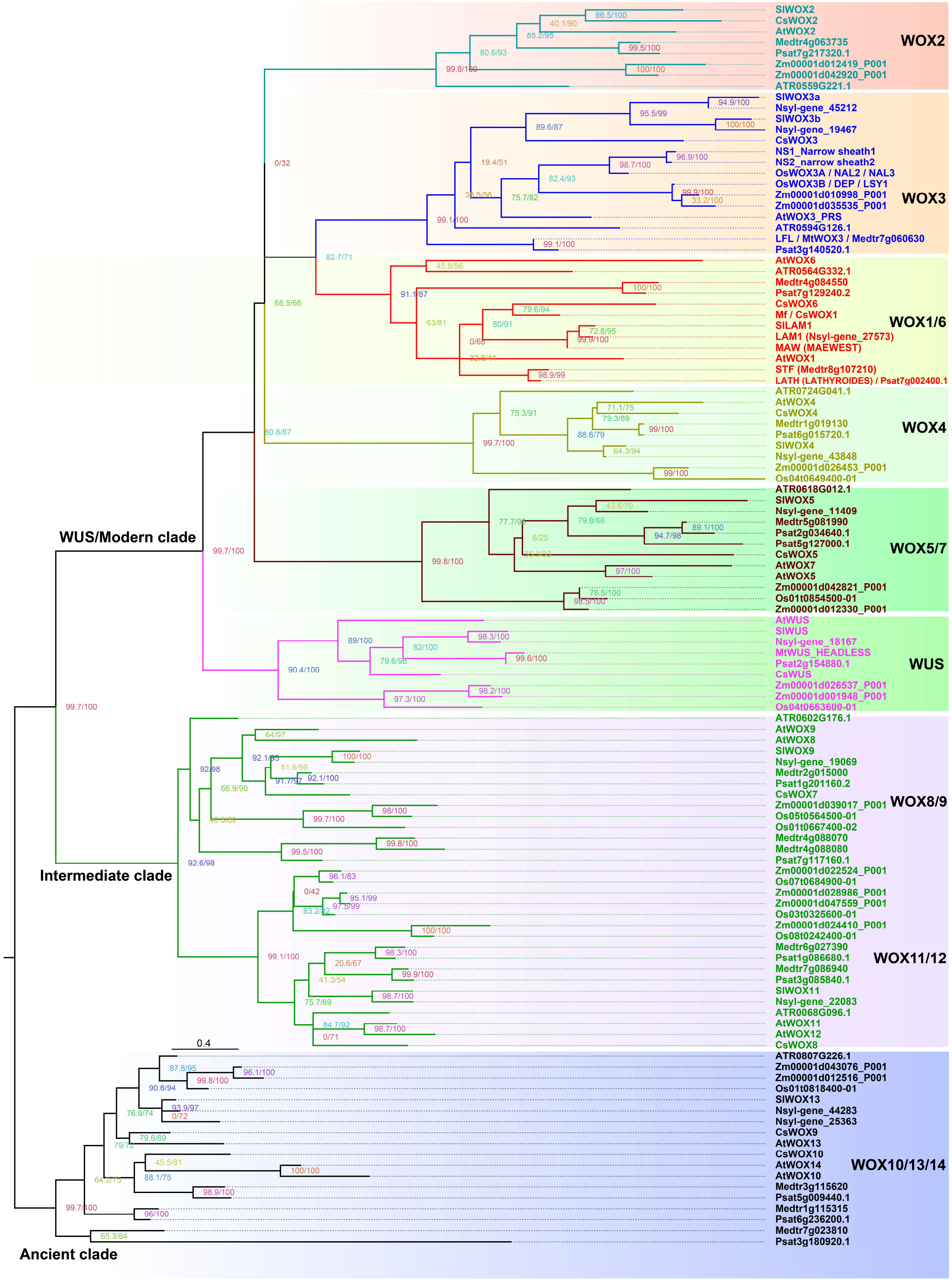


**Fig. S1. Details phylogenetic relationships of the WOX.** WOX proteins from Arabidopsis (At), Medicago (Medtr), tomato (Sl), cucumber (Cs), wild tobacco (Nsyl), rice (Os), maize (Zm), pea (Psat), *Amborella trichopoda* (ATR), and petunia (MAW) were used for phylogenetic analysis.

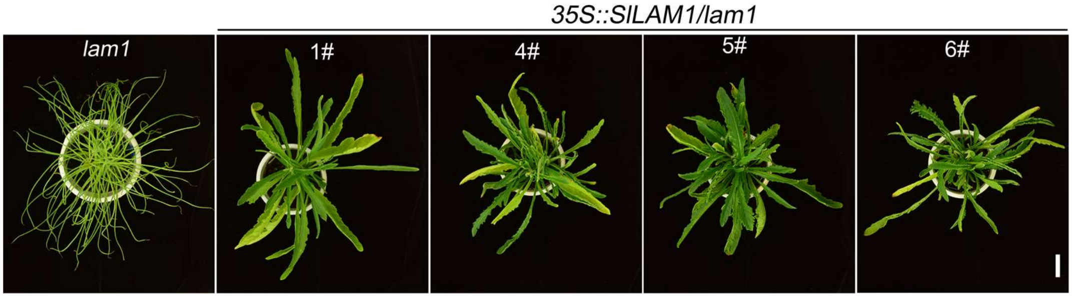


**Fig. S2. Complementation of *lam1* phenotype by *SlLAM1*.** Phenotype of the 4-month-old *lam1* mutant and *35S::SlLAM1/lam1* plants of *N. sylvestris*. *35S:: SlLAM1/lam1* lines displayed expanded but not *lam1*-like leaves, while the non-flowering phenotype of *lam1* were not rescued in these four transgenic lines. Scale bar, 4 cm.

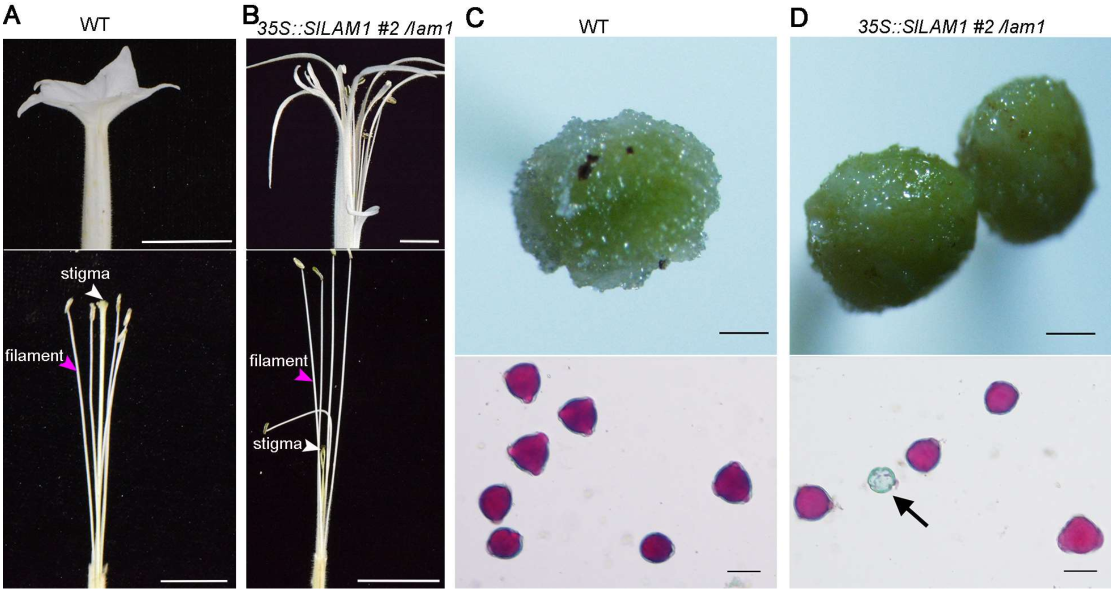


**Fig. S3. Sterility phenotype of *35S::SlLAM1/lam1* plant.** (A-B) Flower organ of WT (A) and a representative *35S::SlLAM1/lam1* plant (B). *35S::SlLAM1/lam1* showed narrower and dehiscent petal (upper panel), and also with visibly shorter style in matured flower compared with filament. White and magenta arrowheads indicate the stigma and filament, respectively. (C-D) Pollen of a representative *35S::SlLAM1/lam1* plant (D, upper panel) was decreased compared with that of WT (C, upper panel). Alexander staining also revealed part of the pollen form *35S::SlLAM1/lam1* plant was aborted (D, lower panel, light blue), being pointed by a black arrow. Non-aborted pollen grains stained in deep pink. Scale bars, 2 cm in (A-B), 200 μm and 10 μm in upper and lower panel of (C-D), respectively.

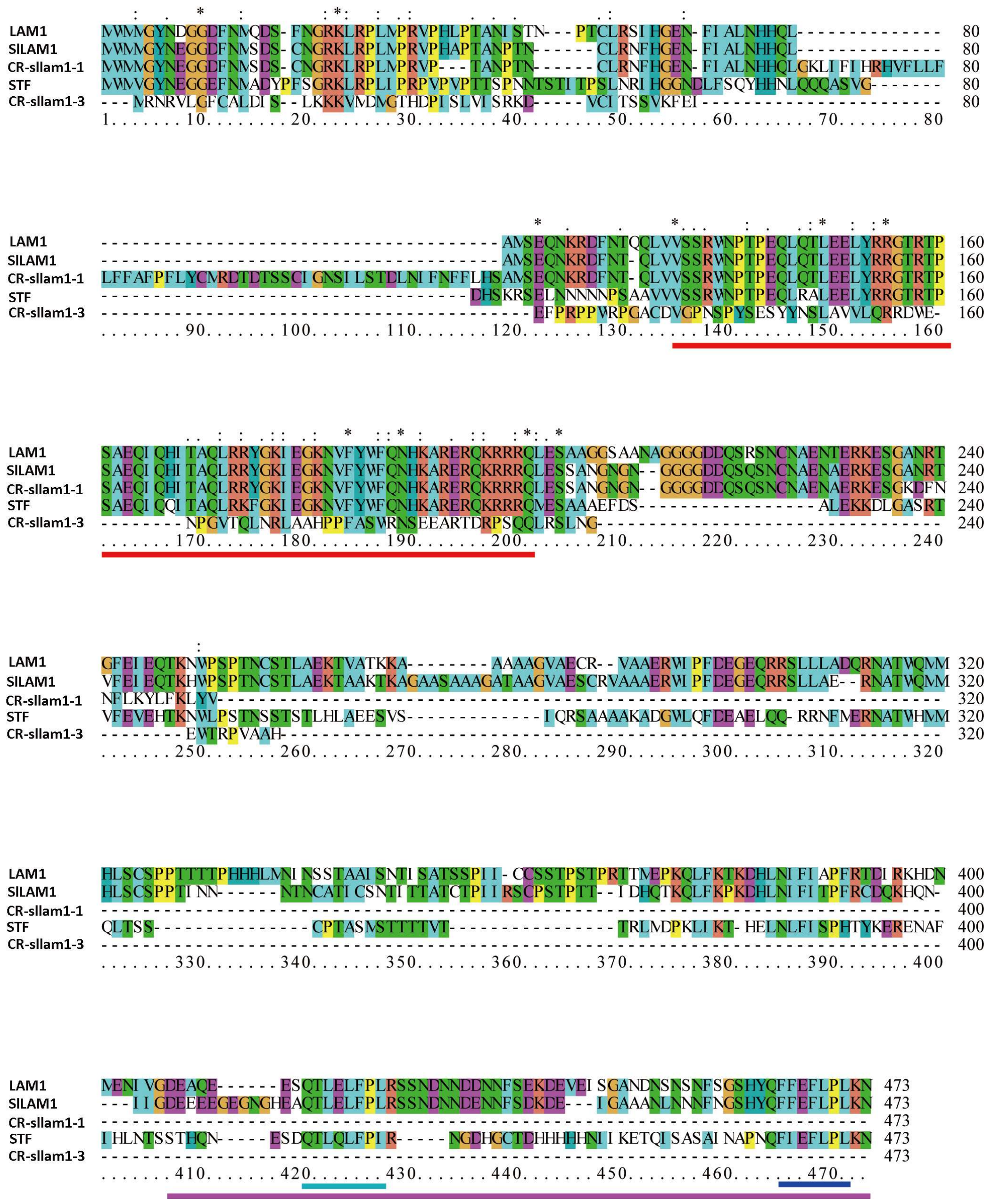


**Fig. S4. Multiple Sequences alignment of STF, LAM1, SlLAM1, and the two CR-sllam1 mutant proteins.** Result of protein sequences alignment of two mutants (including CR-sllam1-1 and CR-sllam1-3), and wild-type STF and SlLAM1. Red line represents homeodomain (HD), purple line represents C-terminal motif containing two motifs: the WUS box (green line) and the STF box (blue). In the *CR-sllam1-3* mutant, MD, HD motif and C-terminal domain were truncated due to the deletion of base pair, and part of the middle domain the C-terminal domain were deleted in the *CR-sllam1-1* mutant.

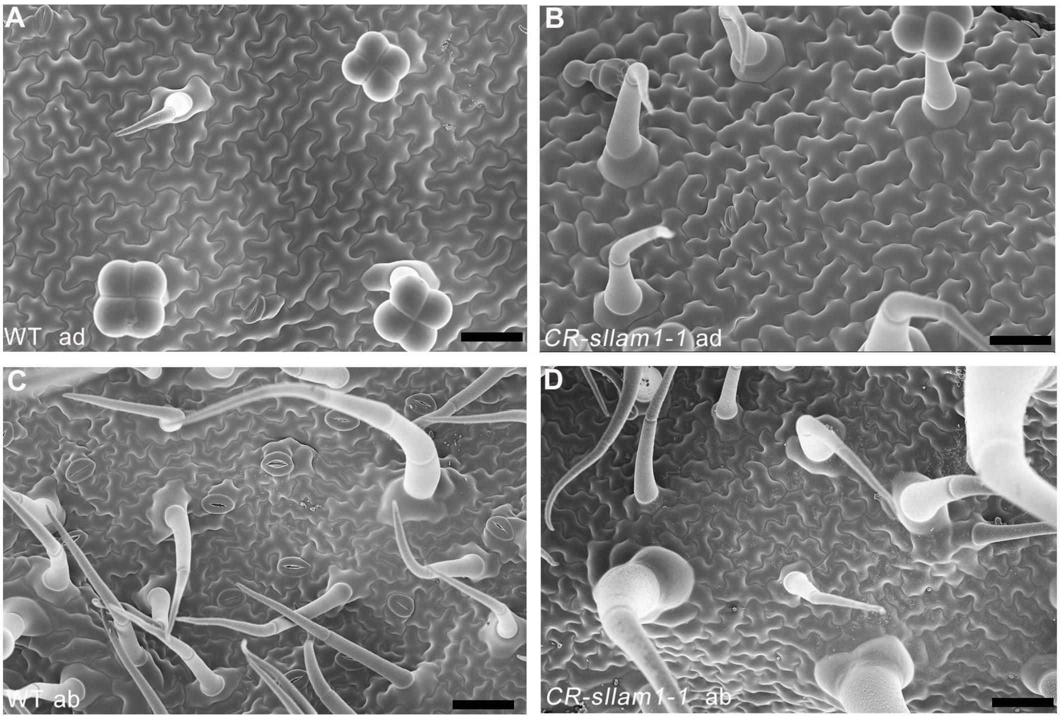


**Fig. S5. Adaxial/abaxial patterning in *CR-sllam1-1* (T1) mutant.** (A-B) SEM analysis of adaxial surface of young leaf from wild-type (A) and *CR-sllam1-1* (T1) mutants (B). (C-D) SEM analysis of abaxial surface of young leaf from wild-type (C) and *CR-sllam1-1* (T1) mutants (D). The morphology of epidermal cells in *CR-sllam1-1* (T1) did not have visible difference compared with that in WT. ad, adaxial surface; ab, abaxial surface. Scale bars, 50 μm in (A-D).

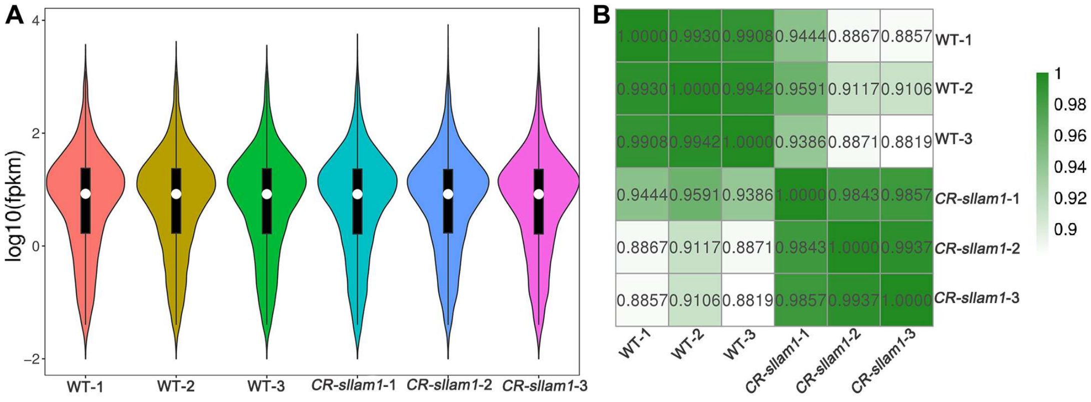


**Fig. S6. Evaluation of reproducibility of RNA-seq experiments.** (A) The violin plot of the overall distribution of FPKM showed that 6 samples have a similar distribution. (B) Heat map showing sample-to-sample Pearson correlation of the normalized gene expression data. Biological replicates cluster well with each other, and samples from WT and *CR-sllam1-1* are clearly separated from each other. Both similar distribution of FPKM and Pearson correlation coefficients greater than 0.98 indicate the high reproducibility of this RNA-seq experiment.

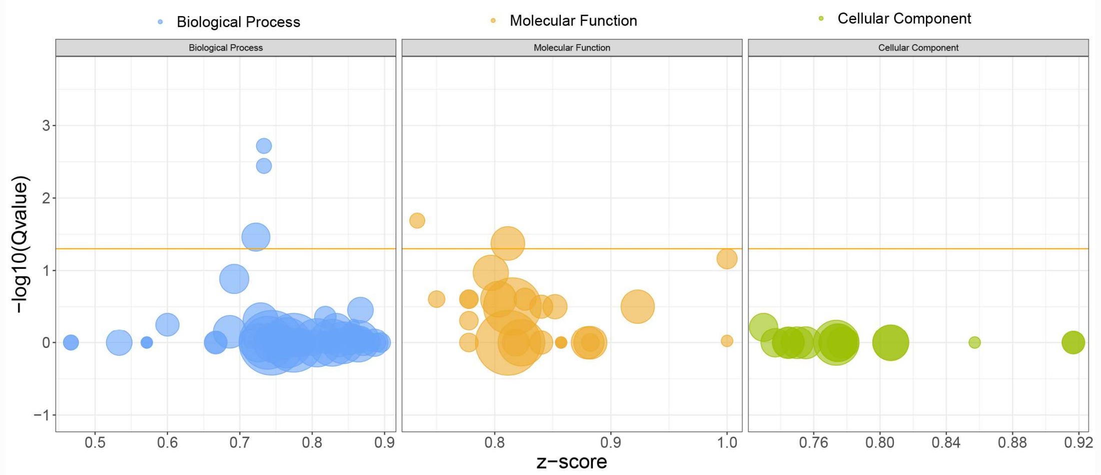


| GO ID | Description |
|---|---|
| GO:0043086 | negative regulation of catalytic activity |
| GO:0044092 | negative regulation of molecular function |
| GO:0010466 | negative regulation of peptidase activity |
| GO:0030162 | regulation of proteolysis |
| GO:0045861 | negative regulation of proteolysis |
| GO:0051346 | negative regulation of hydrolase activity |
| GO:0052547 | regulation of peptidase activity |
| GO:0032269 | negative regulation of cellular protein metabolic process |
| GO:0051248 | negative regulation of protein metabolic |
| GO:0031324 | negative regulation of cellular metabolic |
| GO:0051336 | regulation of hydrolase activity |
| GO:0048523 | negative regulation of cellular process |
| GO:0050790 | regulation of catalytic activity |
| GO:0009892 | negative regulation of metabolic process |
| GO:0065009 | regulation of molecular function |
| GO:0048519 | negative regulation of biological process |
| GO:0010605 | negative regulation of macromolecule metabolic process |
| GO:0008272 | sulfate transport |
| GO:0072348 | sulfur compound transport |
| GO:0015698 | inorganic anion transport |

| GO ID | Description |
|---|---|
| GO:0004857 | enzyme inhibitor activity |
| GO:0004866 | endopeptidase inhibitor activity |
| GO:0030414 | peptidase inhibitor activity |
| GO:0061135 | endopeptidase regulator activity |
| GO:0061134 | peptidase regulator activity |
| GO:0015103 | inorganic anion transmembrane transporter activity |
| GO:0030234 | enzyme regulator activity |
| GO:0001071 | nucleic acid binding transcription factor activity |
| GO:0098772 | molecular function regulator |
| GO:0016705 | oxidoreductase activity, acting on paired donors, with incorporation or reduction of molecular oxygen |
| GO:0015291 | secondary active transmembrane transporter activity |
| GO:0016491 | oxidoreductase activity |
| GO:0016641 | oxidoreductase activity, acting on the CH-NH2 group of donors, oxygen as acceptor |
| GO:0005372 | water transmembrane transporter activity |
| GO:0046906 | tetrapyrrole binding |
| GO:0043169 | cation binding |
| GO:0008509 | anion transmembrane transporter activity |
| GO:0016832 | aldehyde-lyase activity |
| GO:0016638 | oxidoreductase activity, acting on the CH-NH2 |
| GO:0016645 | oxidoreductase activity, acting on the CH-NH |

| GO ID | Description |
|---|---|
| GO:0005576 | extracellular region |
| GO:0071944 | cell periphery |
| GO:0030312 | external encapsulating structure |
| GO:0044421 | extracellular region part |
| GO:0005929 | cilium |
| GO:0031224 | intrinsic component of membrane |
| GO:0031012 | extracellular matrix |
| GO:0042995 | cell projection |
| GO:0033176 | proton-transporting V-type ATPase complex |
| GO:0044425 | membrane part |
| GO:0044459 | plasma membrane part |
| GO:0005886 | plasma membrane |
| GO:0005618 | cell wall |
| GO:0005777 | peroxisome |
| GO:0009528 | plastid inner membrane |
| GO:0016020 | membrane |
| GO:0005911 | cell-cell junction |
| GO:0030054 | cell junction |
| GO:0009532 | plastid stroma |
| GO:0042170 | plastid membrane |

**Fig. S7. GO enrichment plot of DEGs.** FDR-adjusted *p*-value (q value) and z-score were used to plot the GO enrichment (upper panel), and the top 20 subcategories (lower panel) for each ontology were listed.

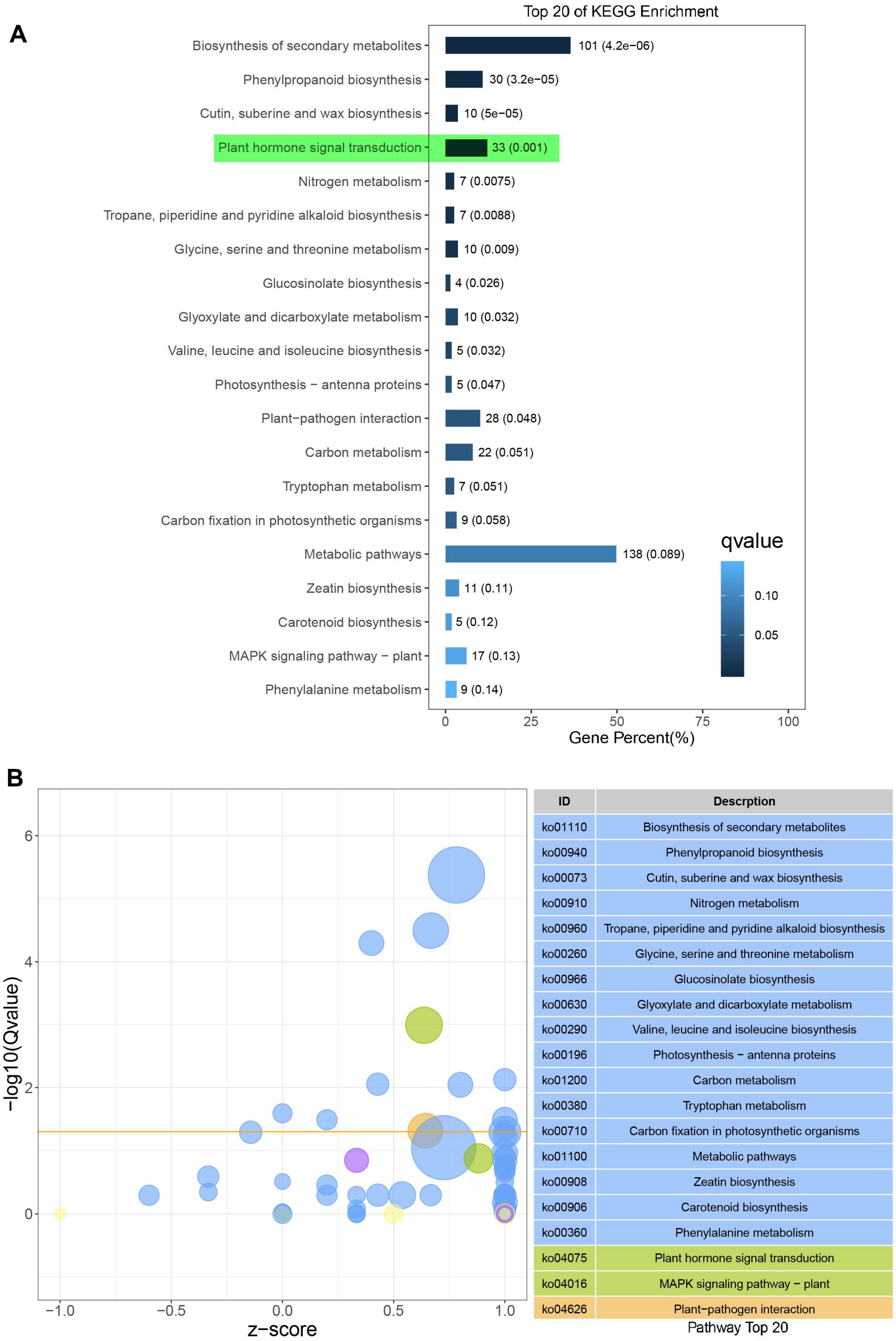


**Fig. S8. KEGG enrichment plot of DEGs.** Bar chart (A) and bubble plot (B) were used to visualize the KEGG pathway enrichment. Both the q-value (A) and z-score (B) were adopted to sort the KEGG pathways, and only the top 20 pathways were shown. Green shading (A) and yellow green circle (B) indicate the enrichment of DEGs in plant hormones related pathway.

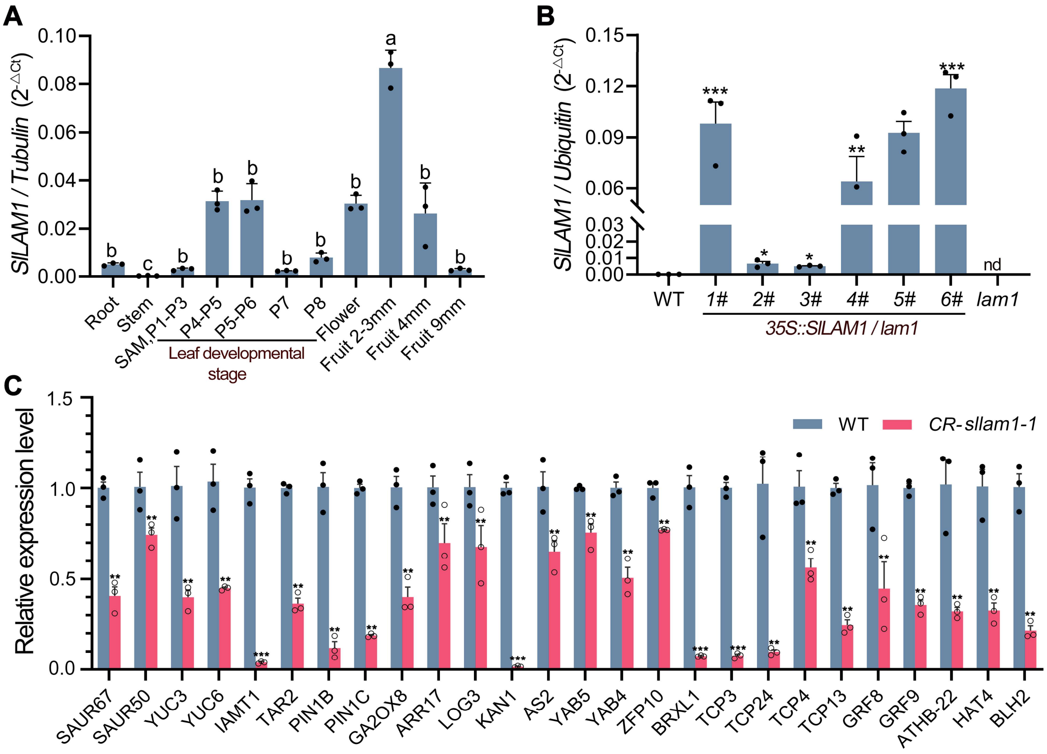


**Fig. S9. RT-qPCR results using alternative reference genes.** Similar RT-qPCR results using alternative reference gene (tomato *Tubulin*) were supplemented for Fig. 2A (A), tobacco *Ubiquitin* gene for Fig. 3C (B), and tomato *Tubulin* for Fig. 7C (C). Different lowercase letters above bars in (A) indicate significant differences of the expression level ($P < 0.05$, n=3), while means sharing the same letter are not significantly different. The asterisks in (B) and (C) denote statistically significant difference between marked samples with corresponding WT (*, $p < 0.05$; **, $p < 0.01$; ***, $p < 0.001$; unpaired two sample *t*-test, n=3). nd, not detected.

**Table. S1. List of primers.**

| Primer name | Sequence (5'- 3') | Usage |
|---|---|---|
| *SlLAM1* (F) | CGAAGAGAAATGTGGATGATGGG | T-cloning vector construction |
| *SlLAM1* (R) | CAGTTCTTGAGTGGAAGGAACTC | |
| *SlLAM1-OE* (F) | TGCTCTAGAATGTGGATGATGGGTTACAA | *p35S::SlLAM1-OE* vector construction |
| *SlLAM1-OE* (R) | CGGGATCCGTTCTTGAGTGGAAGGAACT | |
| *SlLAM1-OE-S (R)* | ATCACCACCACCGCCATTAC | OE sequencing primer |
| *CR-sllam1* T1 (F) | gtcaGGATTCGCCGTAGGCGCATG | *CR-sllam1* vector construction |
| *CR-sllam1* T1 (R) | aaacCATGCGCCTACGGCGAATCC | |
| *CR-sllam1* T2 (F) | gtcaTGTTGGGGATGGCCAGTGCT | |
| *CR-sllam1* T2 (R) | aaacAGCACTGGCCATCCCCAACA | |
| *SlLAM1-IS* | TGTAATACGACTCACTATAGGGCC AGTTCTTGAGTGGAAGGAACTC | RNA *in Situ* Hybridization |
| *CR-sllam1* (F) | GACGACATGCGATGAGAATG | *CR-sllam1* sequencing |
| *CR-sllam1* (R) | CATTCTGATGAATTCCTCAG | |
| *NtActin-A* (F) | CTGGAATGGTTAAGGCTGGA | RT-qPCR of *ACTIN* (GQ281246) for *N. sylvestris* |
| *NtActin -A* (R) | CAATTGCTAACGATTCCGTGT | |
| *NtUbiquitin* (F) | TCCAGGACAAGGAGGGTAT | second reference gene for *N. sylvestris*, RT-qPCR |
| *NtUbiquitin* (R) | CATCAACAACAGGCAACCTAG | |
| *SlACTIN* (F) | GGATCTTGCTGGTCGTGATTT | RT-qPCR of *ACTIN* for *S. lycopersicum* |
| *SlACTIN* (R) | AGTCAAGAGCCACATAGGCA | |
| *SlTubulin* (F) | *AACCTCCATTCAGGAGATGTTT* | second reference gene for *S. lycopersicum,* RT-qPCR |
| *SlTubulin* (R) | *TCTGCTGTAGCATCCTGGTATT* | |
| *SlLAM1-qRT* (F) | AGAACAACTGCAAACGCTGG | qRT-PCR of *SlLAM1* |
| *SlLAM1-qRT* (R) | ACCGTCTAAGTTGTGCAGTGA | |
| *SAUR67-qRT* (F) | GCTCCAACCCAAGGCCTGTC | qRT-PCR *SAUR67* for *CR-sllam1* mutant |
| *SAUR67-qRT* (R) | TCTTGGCGCTGAGCATTGCC | |
| *SAUR50-qRT* (F) | GCCACAAGCAGCAATCTTGA | qRT-PCR *SAUR50* for *CR-sllam1* mutant |
| *SAUR50-qRT* (R) | TGTCCTTTTGGCACGTCGAT | |
| *YUC3-qRT* (F) | CGCGGGCCTTAAACAACAAG | qRT-PCR *YUC3* for *CR-sllam1* mutant |
| *YUC3-qRT* (R) | TCACAGAATTGTCGCGGGAG | |
| *YUC6-qRT* (F) | AAAAGCAATGTGCCCTCGTG | qRT-PCR *YUC6* for *CR-sllam1* mutant |
| *YUC6-qRT* (R) | ATTGAAGCACCAAGCAAGCC | |
| *IAMT1-qRT* (F) | TAGCTTCCAATAGCCACCGC | qRT-PCR *IAMT1-like* for *CR-sllam1* mutant |
| *IAMT1-qRT* (R) | TCCAACACTACATCCGGCAC | |
| *TAR2-qRT* (F) | GACGGATCAATCAGGCAAGC | qRT-PCR *TAR2* in *CR-sllam1* mutant |
| *TAR2-qRT* (R) | CCAGCATGTCCAGTGCATTT | |
| *PIN1B-qRT* (F) | GGACCCTTCGGAAACAACCT | qRT-PCR *PIN1B* for *CR-sllam1* mutant |
| *PIN1B-qRT* (R) | AGGGAGGGTAGAGGTGTACG | |
| *PIN1C-qRT* (F) | CAGTCGGACTTCGAGGTGTC | qRT-PCR *PIN1C* for *CR-sllam1* mutant |
| *PIN1C-qRT* (R) | ATGGACGCCGTATTCCTTGG | |

| | | |
|---|---|---|
| *GA2OX8-qRT* (F) | AGCAGCAATTTTGTTAGCCCC | qRT-PCR *GA2OX8* for *CR-sllam1* mutant |
| *GA2OX8-qRT* (R) | TCCATTGCAGGGTGTGGTTG | |
| *ARR17-qRT* (F) | AAAGCCCTCGACCCTAACTG | qRT-PCR *ARR17* for *CR-sllam1* mutant |
| *ARR17-qRT* (R) | GATGAGCTGCCAAACCCAAA | |
| *LOG3-qRT* (F) | TACCAGGTGGTTATGGCACAC | qRT-PCR *LOG3* for CR-slwox1 mutant |
| *LOG3-qRT* (R) | ATCCTACCGGCTTATCGTGG | |
| *KAN1-qRT* (F) | TGAAGACTCCGAGCAACACA | qRT-PCR *KAN1* for *CR-sllam1* mutant |
| *KAN1-qRT* (R) | TCACGTATGCTGCACCTCTC | |
| *AS2-qRT* (F) | GGAGCAACAACTTCGATGCG | qRT-PCR *AS2* for *CR-sllam1* mutant |
| *AS2-qRT* (R) | ATGGATCAACCGGTGTTCGG | |
| *YAB5-qRT* (F) | GGAAAAGAGGCAACGAGGAC | qRT-PCR *YAB5* for *CR-sllam1* mutant |
| *YAB5-qRT* (R) | TGCCCAATTTTTAGCAGCAGT | |
| *YAB4-qRT* (F) | CGAGCAGCTCTGCTATGTCC | qRT-PCR *YAB4* for *CR-sllam1* mutant |
| *YAB4-qRT* (R) | TGTCCACATCGAACCGTCAC | |
| *ZFP10-qRT* (F) | TGTCGGAGAGAGTTCCGGTC | qRT-PCR *ZFP10* for *CR-sllam1* mutant |
| *ZFP10-qRT* (R) | CGGTGTTTGATTGAGCCTGAC | |
| *BRXL1-qRT* (F) | TTATTCGTCAAGGCCGGGAT | qRT-PCR *BRXL1* for *CR-sllam1* mutant |
| *BRXL1-qRT* (R) | TTCTTGATACCCCACTGCCC | |
| *TCP3-qRT* (F) | TATCGCCACAGCTGATCCAA | qRT-PCR *TCP3* for *CR-sllam1* mutant |
| *TCP3-qRT* (R) | GGCGAATCCGATGCCAGAAA | |
| *TCP24-qRT* (F) | TTTGCCAGCGAAAGCAAAGG | qRT-PCR TCP24 for *CR-sllam1* mutant |
| *TCP24-qRT* (R) | GCTCGCAAACCACATAGCAG | |
| *TCP4-qRT* (F) | CCACAGCAAGGTTTGCACAG | qRT-PCR *TCP4* in *CR-sllam1* mutant |
| *TCP4-qRT* (R) | CTAGCTCATCAATGGCGGGT | |
| *TCP13-qRT* (F) | ACTTCAAGGCAATGGGGAGC | qRT-PCR *TCP13* for *CR-sllam1* mutant |
| *TCP13-qRT* (R) | AGGCCAAGCCTGTCTTGTAG | |
| *GRF8-qRT* (F) | CCATTGGGTGAAGCGTTGTG | qRT-PCR *GRF8* for *CR-sllam1* mutant |
| *GRF8-qRT* (R) | CTCGCAGGAATCAAAGCTGC | |
| *GRF9-qRT* (F) | CCTTGGGTGACGGTGGAATC | qRT-PCR *GRF9* for *CR-sllam1* mutant |
| *GRF9-qRT* (R) | ACATCCCTTTTGCACCTCCA | |
| *ATHB-22-qRT* (F) | AGTCGAAAGCACATCAATGCC | qRT-PCR *ATHB-22* for *CR-sllam1* mutant |
| *ATHB-22-qRT* (F) | GGCCCAATAAGGTGGCATCA | |
| *HAT4-qRT* (F) | GCAAGAAGGGCGATTTGGTC | qRT-PCR *HAT4* for *CR-sllam1* mutant |
| *HAT4-qRT* (R) | TCCCCCATTCTCAAGTCCCA | |
| *BLH2-qRT* (F) | TACCACGGAGTCCATGCAAC | qRT-PCR *BLH2* for *CR-sllam1* mutant |
| *BLH2-qRT* (R) | CTCCGACCGGGCTAGTAAAC | |

**Table. S2. Features of the WOXs proteins in tomato.**

| Gene | Genome ID | Chr | aa | CDS (bp) | Intron/Exon | pI | MW(Da) |
|---|---|---|---|---|---|---|---|
| SlWUS | Solyc02g083950.2.1 | ChrC02 | 272 | 1006 | 2/3 | 6.35 | 31031.16 |
| SlLAM1 | Solyc03g118770.2.1 | ChrC03 | 393 | 1330 | 3/4 | 6.68 | 44057.67 |
| SlWOX2 | Solyc06g076000.1.1 | ChrC06 | 240 | 723 | 1/2 | 8.61 | 26840.07 |
| SlWOX3a | Solyc11g072790.1.1 | ChrC11 | 209 | 630 | 1/2 | 8.77 | 24032.48 |
| SlWOX3b | Solyc11g072770.1.1 | ChrC11 | 197 | 594 | 1/2 | 9.40 | 22760.12 |
| SlWOX4 | Solyc04g078650.2.1 | ChrC04 | 242 | 811 | 2/3 | 8.69 | 27686.05 |
| SlWOX5 | Solyc03g096300.2.1 | ChrC03 | 158 | 493 | 1/2 | 6.32 | 18665.98 |
| SlWOX9 | Solyc02g077390.1.1 | ChrC02 | 358 | 1007 | 2/3 | 7.02 | 40145.49 |
| SlWOX11 | Solyc06g072890.1.1 | ChrC06 | 264 | 795 | 2/3 | 5.38 | 29126.72 |
| SlWOX13 | Solyc02g082670.2.1 | ChrC02 | 274 | 1265 | 2/3 | 5.53 | 30745.37 |

Chr, chromosome; aa, amino acids number; MW, molecular weight.

**Table. S3. RNA-Seq statistics of WT and *CR-sllam1* samples.**

| Organs | Sample | CleanReads | CleanData (bp) | GC (%) | Q30 (%) | UnmappedReads (%) | MappedReads (%) |
|---|---|---|---|---|---|---|---|
| Tomato Shoot buds | WT rep1 | 42892128 | 6.4E+09 | 44.57 | 92.33 | 788762 (1.84%) | 42103366 (98.16%) |
| | WT rep2 | 41405388 | 6.18E+09 | 44.85 | 92.78 | 846946 (2.05%) | 40558442 (97.95%) |
| | WT rep3 | 43342658 | 6.48E+09 | 44.6 | 92.56 | 550164 (1.27%) | 42792494 (98.73%) |
| | *CR-sllam1-1* rep1 | 52912528 | 7.9E+09 | 44.59 | 92.43 | 1138946 (2.15%) | 51773582 (97.85%) |
| | *CR-sllam1-1* rep2 | 40002662 | 5.97E+09 | 44.64 | 92.31 | 452228 (1.13%) | 39550434 (98.87%) |
| | *CR-sllam1-1* rep3 | 45579320 | 6.81E+09 | 44.62 | 92.55 | 691686 (1.52%) | 44887634 (98.48%) |
